# T‐bet Regulates Ion Channels and Transporters and Induces Apoptosis in Intestinal Epithelial Cells

**DOI:** 10.1002/advs.202401654

**Published:** 2024-04-22

**Authors:** Lang Chen, Hongwei Yi, Qingtian Li, Tianhao Duan, Xin Liu, Linfeng Li, Helen Y. Wang, Changsheng Xing, Rong‐Fu Wang

**Affiliations:** ^1^ Center for Inflammation and Epigenetics Houston Methodist Research Institute Houston TX 77030 USA; ^2^ Department of General Surgery Third Xiangya Hospital Xiangya School of Medicine Central South University Changsha 410013 China; ^3^ Department of Pharmacology School of Medicine Southeast University Nanjing 210009 China; ^4^ Department of Medicine Baylor College of Medicine Houston TX 77030 USA; ^5^ Department of Medicine Keck School of Medicine University of Southern California Los Angeles CA 90033 USA; ^6^ Department of Thoracic Surgery Xiangya Hospital Central South University Changsha 410008 China; ^7^ Department of Pediatrics Children's Hospital Los Angeles Keck School of Medicine University of Southern California Los Angeles CA 90027 USA; ^8^ Norris Comprehensive Cancer Center Keck School of Medicine University of Southern California Los Angeles CA 90033 USA

**Keywords:** cancer therapy, cell apoptosis, intestinal epithelial cells, ion channels and transporters, T‐bet

## Abstract

T‐bet, encoded by TBX21, is extensively expressed across various immune cell types, and orchestrates critical functions in their development, survival, and physiological activities. However, the role of T‐bet in non‐immune compartments, notably the epithelial cells, remains obscure. Herein, a Tet‐O‐T‐bet transgenic mouse strain is generated for doxycycline‐inducible T‐bet expression in adult animals. Unexpectedly, ubiquitous T‐bet overexpression causes acute diarrhea, intestinal damage, and rapid mortality. Cell‐type‐specific analyses reveal that T‐bet‐driven pathology is not attributable to its overexpression in CD4^+^ T cells or myeloid lineages. Instead, inducible T‐bet overexpression in the intestinal epithelial cells is the critical determinant of the observed lethal phenotype. Mechanistically, T‐bet overexpression modulates ion channel and transporter profiles in gut epithelial cells, triggering profound fluid secretion and subsequent lethal dehydration. Furthermore, ectopic T‐bet expression enhances gut epithelial cell apoptosis and markedly suppresses colon cancer development in xenograft models. Collectively, the findings unveil a previously unrecognized role of T‐bet in intestinal epithelial cells for inducing apoptosis, diarrhea, and local inflammation, thus implicating its potential as a therapeutic target for the treatment of cancer and inflammatory diseases.

## Graphical abstract

This work presents a novel role of T‐bet in the intestinal epithelial cells. In healthy transgenic mice, induction of T‐bet expression promotes cell apoptosis and regulates ion channel and transporter profiling, leading to diarrhea, tissue damage, and mortality. However, in tumor‐bearing mice, ectopic T‐bet expression effectively inhibits colon cancer development, indicating a promising therapeutic potential in cancer treatment.

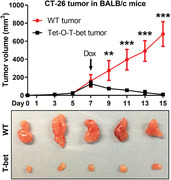

## 1 Introduction

The T‐box transcription factor 21 (TBX21), also known as T‐bet (T‐box‐expressed‐in‐T‐cells), was first identified in 2000 as a pivotal transcription factor for T helper (Th) 1 cell differentiation and subtype‐specific interferon‐γ (IFN‐γ) production.^[^ ^1^ ^]^ T‑bet is extensively expressed across various cell types in both the innate and adaptive immune systems. In the innate immune system, T‑bet is expressed in dendritic cells (DCs), natural killer cells (NKs), natural killer T (NKT) cells, and innate lymphoid cells (ILCs). In the adaptive immune system, T‑bet is expressed in CD4^+^ and CD8^+^ T effector cells, B cells, γδ T cells, and a subset of regulatory T cells (Tregs).^[^ ^2^ ^]^ Overall, T‑bet directs the development of immune cells, regulates their trafficking, and controls the polarity of their cytokine responses. Despite its established immunological functions, the role of T‐bet in other cell or tissue types is rarely documented. A 2005 paper reported the expression of T‐bet in the epithelial cells of the human female reproductive tract, which is modulated by cytokines and female hormones.^[^ ^3^ ^]^ However, besides this report, the role of T‑bet in non‐immune cell types, particularly epithelial cells, remains largely obscure.

T‐bet expression is required for the survival, development, and physiological functions of various immune cell types,^[^ ^2^ ^]^ but its function is multifaceted and context‐dependent. For example, T‐bet is essential for T‐cell differentiation and host immune response to stimulation. Mice with T‐cell‐specific T‐bet overexpression are resistant to experimental autoimmune encephalomyelitis (EAE).^[^ ^4^ ^]^ Furthermore, overexpression of T‐bet in T cells also suppresses autoimmune arthritis development through the dysfunction of Th17 cell differentiation.^[^ ^5^ ^]^ However, in a different autoimmune disease model, the C57BL/6xBXSB/MpJ‐Yaa F1 (Yaa) mice, T‐bet overexpression in T cells exacerbates disease progression and increases mortality rates, with glomerulonephritis and proteinuria.^[^ ^6^ ^]^ Similarly, in other reports, T‐bet overexpression in T cells triggers the spontaneous development of dermatitis and pulmonary alveolar proteinosis, and these mice are hypersensitive to chemical‐induced contact dermatitis.^[^ ^7^ ^]^

The intrinsic correlation between T‐bet and intestinal inflammation has been well acknowledged.^[^ ^8^ ^]^ T‐bet expression is relatively low in the intestinal epithelia under homeostatic condition but dramatically increased during enteritis. In human ulcerative colitis patients, T‐bet is significantly increased based on genome‐wide gene profiling.^[^ ^9^ ^]^ Similarly, T‐bet elevation was observed in human Crohn's disease patients in both inflamed colons and small intestines.^[^ ^10^ ^]^ Consistently, in dextran sodium sulfate (DSS)‐treated mouse colitis and azoxymethane (AOM)/DSS‐induced colon cancer models, whole genome expression profiling showed the upregulation of T‐bet in colorectal epithelia.^[^ ^11^ ^]^ These sequencing data show the upregulation of T‐bet under pathological conditions, but whether T‐bet is highly expressed in gut epithelial cells or tissue‐infiltrated immune cells remains to be elucidated.

In human Crohn's disease and celiac disease patients, elevated expression of T‐bet has been confirmed in lamina propria T cells, particularly Th1 cells as potential pathological effectors, as well as peripheral blood T cells, B cells, and monocytes.^[^ ^12^ ^]^ T‐bet levels tend to decrease in these immune cells after the disease remission, while anti‐inflammatory therapies for IBD, such as glucocorticoids, inhibit T‐bet expression in T cells.^[^ ^12^ ^]^ In preclinical models, the intestinal balance between IFN‐γ/IL‐4 and transforming growth factor (TGF)‐β activity is the critical determinant to control intestinal inflammation. Overexpression of T‐bet is essential and sufficient to promote Th1‐mediated colitis by controlling the balance of IFN‐γ/IL‐4 and TGF‐β responses, T cell activation, and disease progression.^[^ ^8^ ^]^ Furthermore, under the stimulation of segmented filamentous bacteria (SFB) and *Helicobacter hepaticus*, T‐bet‐deficient Th cells can differentiate into Th1/17 cells, which coexpress IFN‐γ and interleukin (IL)‐17 and efficiently induce colitis.^[^ ^13^ ^]^ While IL‐17A neutralization exacerbated the colitis induced by T‐bet‐deficient Th cells, IFN‐γ neutralization completely abolished the disease,^[^ ^13^ ^]^ indicating that T‐bet‐mediated Th1/Th17 balance is also important for controlling colitis. Also, T‐bet directly activates Th1‐specific chemokine receptors, such as CXCR3 and CCR5, which are critical for homing to inflamed tissues.^[^ ^2^ ^]^ In the innate immune system, T‐bet deficiency controls the response of the mucosal immune system to commensal bacteria and increases susceptibility to colitis.^[^ ^14^ ^]^ Furthermore, T‐bet is a central transcriptional regulator for intraepithelial lymphocyte (IEL) development and controls susceptibility to chemically induced colitis.^[^ ^15^ ^]^ Besides in these immune cells, the function of T‐bet in intestinal epithelial cells has not been reported.

To investigate the in vivo function of T‐bet beyond the immune landscape, we generated a Tet‐O‐T‐bet transgenic mouse model for doxycycline‐inducible T‐bet expression. Unexpectedly, systemic T‐bet overexpression causes acute diarrhea, intestinal tissue damage, and rapid mortality in 2–4 days. Interestingly, using cell‐type‐specific T‐bet expressing mice, we found that T‐bet overexpression in CD4^+^ T cells or myeloid cells did not cause the animal death. Instead, inducible T‐bet overexpression in the intestinal epithelial cells was the critical determinant of the lethal phenotype. Mechanistically, we showed that T‐bet overexpression modulated ion channel and transporter profiles in gut epithelial cells, which in turn triggered fluid secretion into small intestine and subsequent lethal dehydration. Furthermore, ectopic T‐bet expression directly induced gut epithelial cell apoptosis and markedly suppressed colon cancer development in xenograft models. Collectively, our findings provide novel insight into the mechanism by which T‐bet regulates ion channel and transporter expression and induces apoptosis in gut epithelial cells, thus identifying a potential therapeutic target for the treatment of cancer and inflammatory diseases.

## 2 Results

### 2.1 Systemic Induction of T‐bet Overexpression Leads to Severe Diarrhea and Mortality in Mice

T‐bet is a pivotal transcription factor has been extensively studied in various immune cell populations, yet function of T‐bet in non‐immune cells are rarely documented. To evaluate the expression levels of T‐bet (encoded by TBX21) in normal human tissues, we performed the “Body Atlas” analysis using BaseSpace Correlation Engine 2.0 from Illumina Inc. Among all 147 tested human tissue types from the database, we found the expression levels of T‐bet were high in spleen, heart, and kidney; intermediate in liver, lung, and digestive tissues; and low in nervous system and glands (Figure S1, Supporting Information). In immune system, T‐bet was highly expressed in peripheral CD4 and CD8 T cells, but had a low expression in Treg cells and macrophages (Figure S1, Supporting Information).

To study the in vivo function of T‐bet, we generated the Tet‐O‐T‐bet transgenic mouse strain by incorporating the mouse *Tbx21* coding sequence into a doxycycline‐inducible expression system (Figure S2a, Supporting Information). The Tet‐O‐T‐bet mouse strain was then crossed with the R26‐M2rtTA (systemic rtTA expression) strain, and double‐positive Tet‐O‐T‐bet:rtTA mice were generated for further experiments (Figure S2b, Supporting Information). The induction of T‐bet expression in different tissues was evaluated in four mice at early generation, after doxycycline (Dox) treatment. Although the expression levels varied, T‐bet was induced in all the tested organs and tissues from the transgenic mice, including lymph nodes (LNs), thymus, skin, spleen, lung, liver, and purified splenocytes (Figure S2c, Supporting Information). Since T‐bet is a key regulator in CD8^+^ and Th1 CD4^+^ T cells, we evaluated the T cell populations in lymphoid organs from Tet‐O‐T‐bet:rtTA mice, and found that doxycycline‐induced T‐bet overexpression enhanced the differentiation of both CD4^+^ and CD8^+^ T cells in the thymus and spleen (Figure S2d, Supporting Information). CD8^+^ T cell population was also increased in the LNs (Figure S2d, Supporting Information). These observations are consistent with previous reports that T‐bet promotes the differentiation of both CD4^+^ and CD8^+^ T cells.^[^ ^16^ ^]^ We further validated the induction of T‐bet expression by flow cytometry, and observed a rapid increase of T‐bet^+^ cells after the first day of doxycycline treatment in gated CD4^+^ lymphocytes (Figure S2e, Supporting Information).

**Figure 1:**
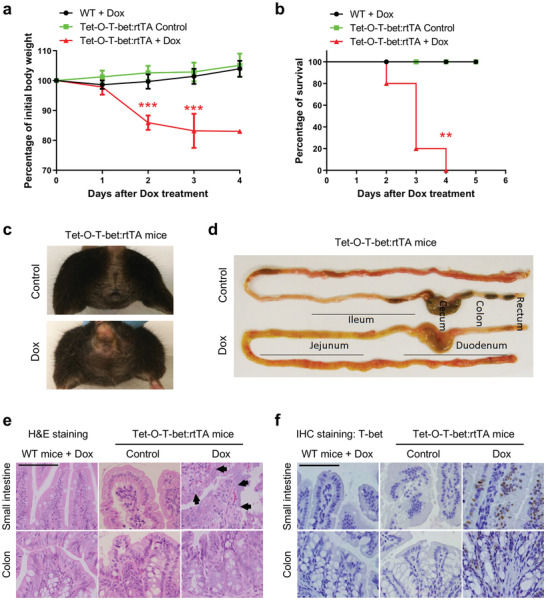
Systemic induction of T‐bet overexpression leads to severe diarrhea and mortality in mice. a,b) WT and Tet‐O‐T‐bet:rtTA (systemic T‐bet overexpression) mice were continuously treated with control water (5% sucrose alone) or doxycycline water solution (1 mg mL^−1^ in 5% sucrose). a) Body weights (mean ± SD) and b) survival curves were observed after the treatment (*n* = 5). c–f) Tet‐O‐T‐bet:rtTA mice were treated with control water or doxycycline water solution for 2 days. c) On day 2, severe diarrhea can only be observed in Dox‐treated mice, based on the hair, structure, and wetness of the anus area. d) Mice were euthanized on day 2 and whole intestines were dissected to display the morphological change after Dox‐induced T‐bet overexpression. e) H&E staining on intestinal sections to show tissue structure in control and T‐bet overexpressed mice. Arrows were added to indicate the tissue damage in the small intestine of Dox‐treated Tet‐O‐T‐bet:rtTA mice (scale bar: 200 µm). f) IHC staining of T‐bet on intestinal sections to validate the Dox‐induced T‐bet overexpression (scale bar: 200 µm). Statistical analyses: a) ANOVA and student's unpaired t test and b) Mantel‐Cox log‐rank test. **p < 0.01; ***p < 0.001.

Next, we observed the animal condition and unexpectedly found that Dox‐treated Tet‐O‐T‐bet:rtTA mice had significantly decreased body weights and died within 4 days (**Figure** **1**a,b). On the contrary, Dox‐treated wild‐type (WT) mice and control water‐treated Tet‐O‐T‐bet:rtTA mice all survived with stable body weights (Figure 1a,b). Furthermore, after doxycycline‐induced overexpression of T‐bet, severe diarrhea was observed in Tet‐O‐T‐bet:rtTA mice (Figure 1c), indicating the potential dehydration in these mice that was consistent with the decreased body weight after doxycycline treatment. The intestines were further dissected for morphological analysis, and we found that the intestines of Dox‐treated Tet‐O‐T‐bet:rtTA mice were swollen and empty, without solid fecal materials but full of yellowish fluids, indicating diarrhea and gut inflammation (Figure 1d). Major organs and tissues were collected from control and T‐bet‐expressing mice for histopathological analyses after hematoxylin and eosin (H&E) staining. We found that Dox‐treated Tet‐O‐T‐bet:rtTA mice had severely destroyed gut epithelia in the small intestine (Figure 1e), while most of other organs remained unaffected (Figure S3a, Supporting Information). We also validated T‐bet overexpression by Immunohistochemistry (IHC) staining, and observed the successful induction of T‐bet expression in the liver, spleen, lung, kidney, thymus, LNs, stomach, and intestine from Dox‐treated Tet‐O‐T‐bet:rtTA mice (Figure 1f; Figure S3b, Supporting Information). These data collectively demonstrate that systemic overexpression of T‐bet is lethal to mice, probably due to severe diarrhea and intestinal tissue damage.

### 2.2 Cell‐Specific Induction of T‐bet Overexpression in CD4^+^ T Cells or Myeloid Lineage Does Not Elicit Mouse Mortality

To ascertain the specific cell subset responsible for T‐bet‐induced mouse mortality, we generated a cell‐type specific T‐bet expression system by crossing Tet‐O‐T‐bet mice with the rtTA‐EGFP strain, in which the LoxP‐flanked stop sequence was inserted in front of EGFP‐tagged rtTA. After crossing with cell‐type‐specific Cre mice, the stop sequence will be removed, and rtTA will be expressed in Cre‐expressing cells to induce cell‐specific T‐bet expression (Figure S4a, Supporting Information).

**Figure 2:**
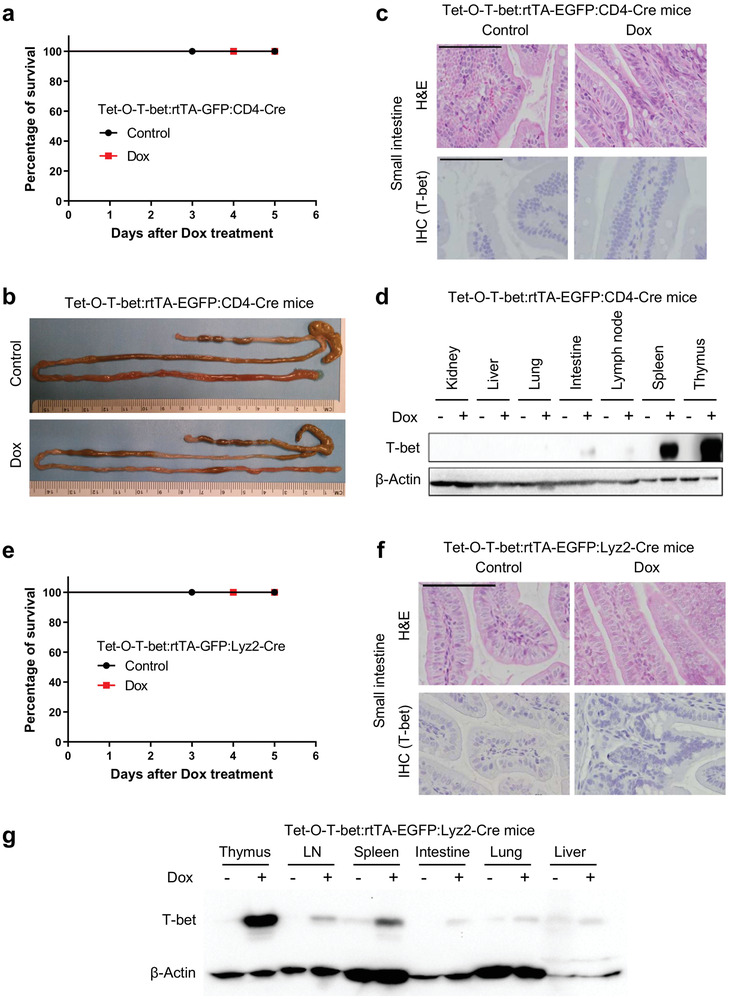
Cell‐specific induction of T‐bet overexpression in CD4^+^ T cells or myeloid lineage does not elicit mouse mortality. a) Tet‐O‐T‐bet:rtTA‐GFP:CD4‐Cre (specific T‐bet overexpression in CD4 cells) mice were continuously treated with control water (5% sucrose alone) or doxycycline water solution (1 mg mL^−1^ in 5% sucrose). Survival curves were observed after the treatment (*n* = 3). b–d) Tet‐O‐T‐bet:rtTA‐GFP:CD4‐Cre mice were treated with control water or doxycycline water solution for 2 days. b) Mice were euthanized on day 2 and whole intestines were dissected to display the morphology. c) H&E staining and IHC staining of T‐bet on small intestine sections to show tissue structure and T‐bet overexpression (scale bar: 200 µm). d) Overexpression of T‐bet in selected tissues and organs by western blotting. e) Tet‐O‐T‐bet:rtTA‐GFP:Lyz2‐Cre (specific T‐bet overexpression in myeloid cells such as macrophages and neutrophils) mice were continuously treated with control water or doxycycline water solution. Survival curves were observed after the treatment (*n* = 4). f,g) Tet‐O‐T‐bet:rtTA‐GFP:Lyz2‐Cre mice were treated with control water or doxycycline water solution for 2 days. f) H&E staining and IHC staining of T‐bet on small intestine sections to show tissue structure and T‐bet overexpression (scale bar: 200 µm). g) Overexpression of T‐bet in selected tissues and organs by western blotting. Statistical analyses: Mantel‐Cox log‐rank test (a,e).

First, we generated the Tet‐O‐T‐bet:rtTA‐GFP:CD4‐Cre mice (Figure S4b, Supporting Information), in which T‐bet expression could be induced by doxycycline in CD4^+^ T cells. The induction of T‐bet expression in T cells, but not in B cells (as a negative control), was validated by flow cytometry and western blotting in sorted splenocytes from these mice (Figure S4c,d, Supporting Information). Next, we treated the Tet‐O‐T‐bet:rtTA‐GFP:CD4‐Cre mice with doxycycline, and found these mice remained alive (**Figure** **2**a) and were all under a healthy condition (data not shown). The whole intestines were collected for morphological analysis, and we did not observe any differences between control and T‐bet overexpressed mice (Figure 2b). H&E staining was also performed on the intestine sections and other major tissues, and no pathological alteration was observed (Figure 2c; Figure S4e, Supporting Information). T‐bet expression levels in different organs were also tested by IHC staining and western blotting. After doxycycline treatment, T‐bet was highly induced in lymphoid organs (such as thymus, spleen, and LNs), and could also be detected in tissues with frequent T‐cell infiltration (such as lung and intestine), but not other tissue types (Figure 2c,d; Figure S4e, Supporting Information).

In parallel, we generated the Tet‐O‐T‐bet:rtTA‐GFP:Lyz2‐Cre mice (Figure S5a, Supporting Information) to specifically induce T‐bet expression in myeloid lineages, such as macrophages and neutrophils. The Dox‐induced expression of T‐bet in CD11b^+^ myeloid population was validated in myeloid‐cell‐enriched bone marrow samples (Figure S5b, Supporting Information). After doxycycline treatment, Tet‐O‐T‐bet:rtTA‐GFP:Lyz2‐Cre mice still survived (Figure 2e) and were under a healthy condition (data not shown). H&E staining further showed that there was no pathological alteration in the small intestine or other major organs from these mice (Figure 2f; Figure S5c, Supporting Information). Tissue‐specific T‐bet overexpression in Dox‐treated Tet‐O‐T‐bet:rtTA‐GFP:Lyz2‐Cre mice was also confirmed by western blotting and IHC staining (Figure 2f,g; Figure S5d, Supporting Information). Taken together, these data rule out that the diarrhea and mortality observed in systemic T‐bet‐expressing mice are due to the enhanced T‐bet expression in CD4^+^ T cells or myeloid lineage.

### 2.3 Immune Cells Are Not the Principal Mediators of T‐bet Overexpression‐Induced Mortality

**Figure 3:**
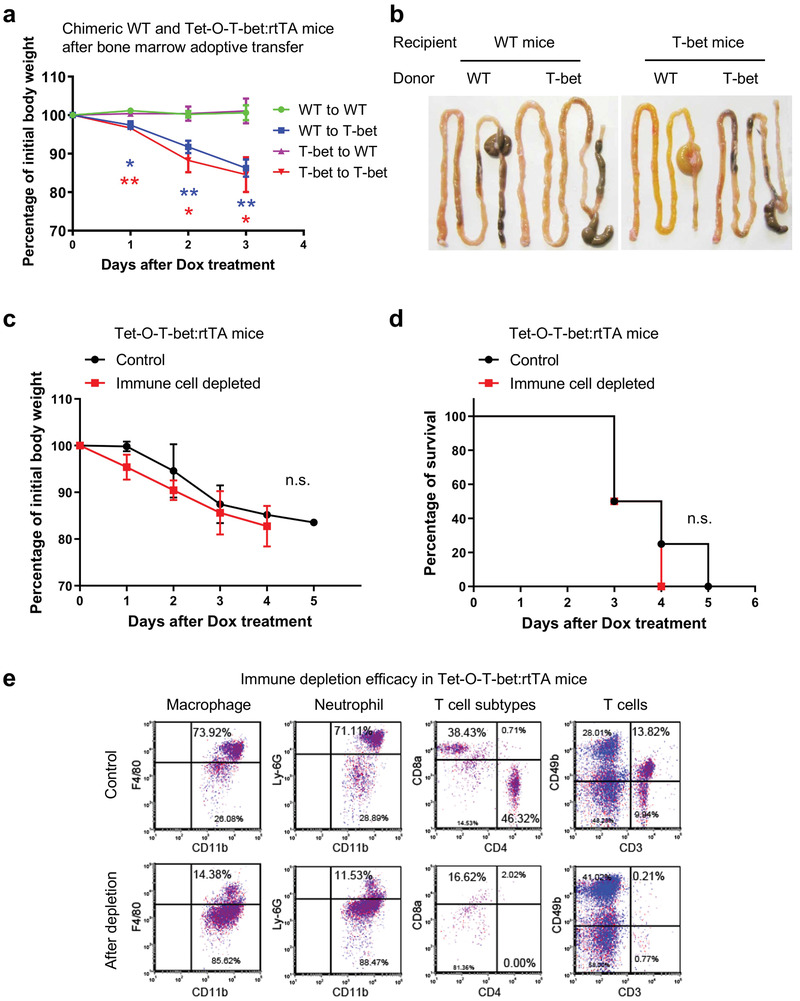
Immune cells are not the principal mediators of T‐bet overexpression‐induced mortality. a,b) WT and Tet‐O‐T‐bet:rtTA recipient mice were irradiated and transferred with bone marrow from donor WT and Tet‐O‐T‐bet:rtTA mice. The chimeric mice were continuously treated with doxycycline water solution (1 mg mL^−1^ in 5% sucrose). a) Changes on the body weights after the doxycycline treatment (mean ± SD) (*n* = 3). b) Whole intestines from the chimeric mice to display the morphology. c–e) Tet‐O‐T‐bet:rtTA mice were treated with clodronate liposomes, Ly‐6G antibody (Ab), CD4 Ab, and CD8 Ab to simultaneously deplete macrophages, neutrophils, and T cells. PBS‐control liposomes and isotype‐control Abs were used in control group. Mice were continuously treated with doxycycline water solution. The c) body weight changes (mean ± SD) and d) survival curves were observed and analyzed (*n* = 4). e) Before the doxycycline treatment, blood samples were collected to validate the cell depletion efficacy by flow cytometry analyses in gated CD45^+^/CD11b^+^ myeloid cells and CD45^+^/CD3^+^ lymphocytes. The percentages of macrophages (73.92% vs 14.38%), neutrophils (71.11% vs 11.53%), CD4^+^ T cells (46.32% vs 0%), CD8^+^ T cells (38.43% vs 16.62%), CD3^+^ T cells (9.94% vs 0.77%), and NKT cells (13.82% vs 0.21%) were all dramatically decreased after the cell depletion. Statistical analyses: ANOVA and student's unpaired t test (a,c) and d) Mantel‐Cox log‐rank test. *p < 0.05; **p < 0.01.

To dissect the role of immune versus non‐immune cells (such as epithelial cells) in T‐bet‐mediated mouse mortality, we performed bone marrow adoptive transfer and generated chimeric mice with altered immune cell genotypes. After doxycycline treatment, we found that the WT recipient mice all maintained healthy with no change in body weights, regardless of the donor's genotype (**Figure** **3**a). In contrast, Tet‐O‐T‐bet:rtTA recipient mice with either WT or T‐bet^+^ immune cells showed significantly declined body weights and conditions compared to the WT recipients (Figure 3a). The morphology of intestine also revealed severe gut inflammation in the Tet‐O‐T‐bet:rtTA recipient mice, as they were swollen and lacked solid fecal materials (Figure 3b), mirroring the phenotype of systemic T‐bet‐overexpressing mice.

Furthermore, we simultaneously depleted T cells and myeloid cells in Tet‐O‐T‐bet:rtTA mice to determine whether loss of these key T‐bet‐expressing immune populations may alter the mouse phenotype. We found that the simultaneous depletion of these immune cell types could not improve body weight loss and mouse survival in Dox‐treated Tet‐O‐T‐bet:rtTA mice (Figure 3c,d). Successful depletion of these immune cells was validated by flow cytometry analysis of the blood samples (Figure 3e). These data suggest that immune cells are not the key contributors to T‐bet‐induced diarrhea and mortality, indicating the significance of other cell types, such as epithelial cells, in this process.

### 2.4 Gut Epithelial Cell‐Specific T‐bet Overexpression Drives Mouse Mortality

Given the observations of gastrointestinal symptoms in Tet‐O‐T‐bet:rtTA mice, such as diarrhea, gut inflammation, and epithelial tissue damage, we hypothesize that intestinal epithelial cells play an important role in T‐bet‐induced mouse mortality. Therefore, we crossed Tet‐O‐T‐bet:rtTA‐GFP mice with Villin‐Cre mice, and generated the Tet‐O‐T‐bet:rtTA‐GFP:Villin‐Cre strain (Figure S6a, Supporting Information) to specifically induce T‐bet overexpression in gut epithelial cells.

**Figure 4:**
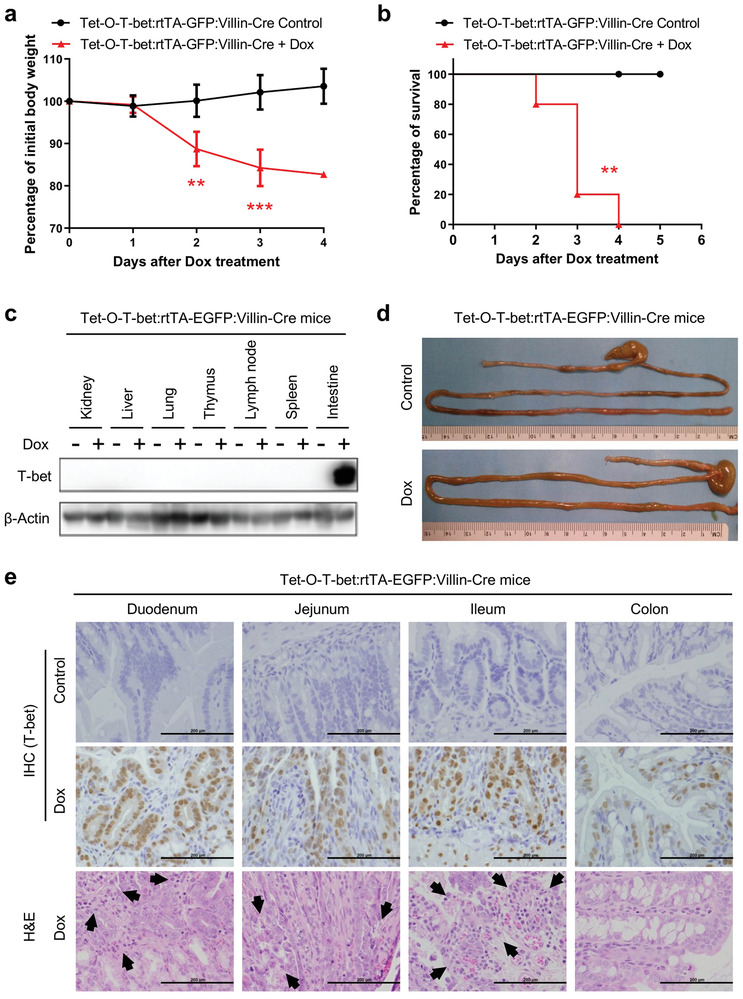
Gut epithelial cell‐specific T‐bet overexpression drives mouse mortality. a,b) Tet‐O‐T‐bet:rtTA‐GFP:Villin‐Cre (specific T‐bet overexpression in gut epithelial cells) mice were continuously treated with control water (5% sucrose alone) or doxycycline water solution (1 mg mL^−1^ in 5% sucrose). The a) body weight changes (mean ± SD) and b) survival curves were observed and analyzed (*n* = 5). c–e) Tet‐O‐T‐bet:rtTA‐GFP:Villin‐Cre mice were treated with control water or doxycycline water solution for 2 days. Mice were euthanized on day 2 for tissue collection. c) Overexpression of T‐bet in selected tissues and organs by western blotting. d) whole intestines were dissected to display the morphological change after Dox‐induced T‐bet overexpression. e) IHC staining of T‐bet on intestinal sections to validate the Dox‐induced T‐bet overexpression. H&E staining on intestinal sections to show tissue structure in control and T‐bet overexpressed mice. Arrows were added to indicate the tissue damage in the small intestine of Dox‐treated Tet‐O‐T‐bet:rtTA‐GFP:Villin‐Cre mice (scale bar: 200 µm). Statistical analyses: a) ANOVA and student's unpaired t test and b) Mantel‐Cox log‐rank test. **p < 0.01; ***p < 0.001.

After treatment with doxycycline, the body weights of Tet‐O‐T‐bet:rtTA‐GFP:Villin‐Cre mice decreased significantly compared to the control group (**Figure** **4**a). Consistent with the phenotype of systemic T‐bet‐overexpressing mice, Tet‐O‐T‐bet:rtTA‐GFP:Villin‐Cre mice died within 4 days after Dox‐induced T‐bet expression (Figure 4b). Western blotting data validated the specific expression of T‐bet in the intestine but not in other organs from the Tet‐O‐T‐bet:rtTA‐GFP:Villin‐Cre mice (Figure 4c). Consistently, we observed morphological differences in the intestine between the control and Dox‐treated Tet‐O‐T‐bet:rtTA‐GFP:Villin‐Cre mice, which showed gut inflammation and signs of diarrhea after the induction of T‐bet (Figure 4d). Notably, most of the intestinal damage was observed after day 2, and no significant damage was found on the first day of doxycycline treatment (Figure S6b, Supporting Information). Furthermore, H&E staining confirmed the gut epithelial damage in the small intestine of Dox‐treated Tet‐O‐T‐bet:rtTA‐GFP:Villin‐Cre mice (Figure 4e). The induction of T‐bet overexpression was validated by IHC staining in all the small intestine sections and the colon (Figure 4e). As expected, we did not observe either T‐bet expression or tissue abnormality in other major organs from Dox‐treated Tet‐O‐T‐bet:rtTA‐GFP:Villin‐Cre mice (Figure S6c, Supporting Information). Taken together, these data highlight the potent and specific pathophysiological impact of T‐bet overexpression in gut epithelial cells in triggering diarrhea and mouse mortality.

### 2.5 T‐bet Modulates Ion Channel and Transporter Expression in Intestinal Epithelial Cells

The ion channels and transporters in intestinal epithelial cells are crucial for maintaining the water‐electrolyte balance and acid‐base homeostasis, thus directly controlling water secretion and absorption in gut.^[^ ^17^ ^]^ Since we observed severe mouse diarrhea and dehydration after inducing T‐bet overexpression in gut epithelial cells, we further investigated the expression levels of ion channels and transporters. Among the tested candidates, cystic fibrosis transmembrane conductance regulator (CFTR) and Anoctamin‐1 (ANO1, also known as Transmembrane member 16A, or TMEM16A) are the most important chloride channels for chloride efflux, promoting water secretion from the cells.^[^ ^18^ ^]^ In contrast, the solute carriers (SLCs) family members, including SLC5A1 (or SGLT1), SLC9A3 (or NHE3), SLC26A3 (or DRA), and SLC26A6, are ion transporters for chloride and sodium ion uptake, promoting water absorption into the cells.^[^ ^18^, ^19^ ^]^

**Figure 5:**
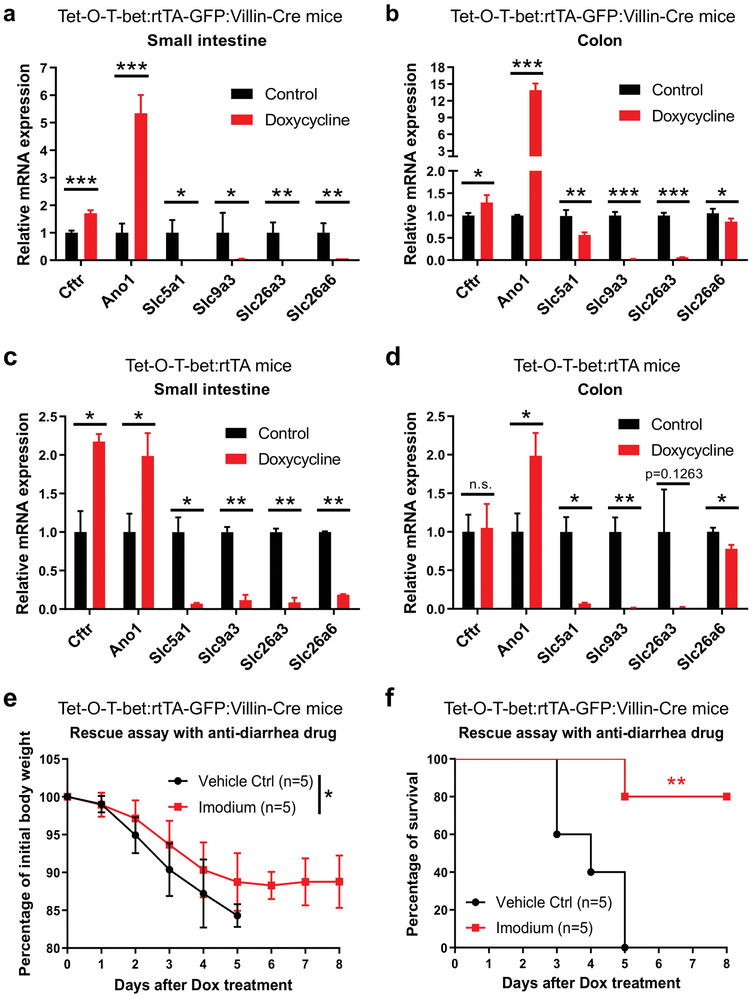
T‐bet modulates ion channel and transporter expression in intestinal epithelial cells. a,b) Tet‐O‐T‐bet:rtTA‐GFP:Villin‐Cre mice (gut epithelia specific T‐bet overexpression) were treated with control water (5% sucrose alone) or doxycycline water solution (1 mg mL^−1^ in 5% sucrose) for 2 days. Mice were euthanized on day 2, and RNA samples were isolated from intestinal tissues. Real‐time QPCR was performed to detect the expression levels of key ion channels and transporters in a) the small intestine and b) colon (mean ± SD). c,d) Tet‐O‐T‐bet:rtTA mice (systemic T‐bet overexpression) were treated with control water or doxycycline water solution for 2 days. Mice were euthanized on day 2, and RNA samples were isolated from intestinal tissues. Real‐time QPCR was performed to detect the expression levels of key ion channels and transporters in c) the small intestine and d) colon (mean ± SD). e,f) Tet‐O‐T‐bet:rtTA‐GFP:Villin‐Cre mice were orally treated with PBS control or anti‐diarrhea drug Imodium (125 µg Loperamide hydrochloride in 200 µL PBS) every day since day ‐1. Doxycycline water solution was continuously treated since day 0. The e) body weight changes (mean ± SD) and f) survival curves were observed and analyzed (*n* = 5). Statistical analyses: a–d) Student's unpaired t test, e) ANOVA and student's unpaired t test, and f) Mantel‐Cox log‐rank test. *p < 0.05; **p < 0.01; ***p < 0.001.

We treated the Tet‐O‐T‐bet:rtTA‐GFP:Villin‐Cre mice with control water or doxycycline water solution, and euthanized the mice on day 2 for RNA samples from intestinal tissues. Real‐time quantitative PCR (qPCR) analyses revealed significantly increased expression of Cftr and Ano1 in both small intestine and colon tissues after the induction of T‐bet overexpression (**Figure** **5**a,b). Meanwhile, the expression levels of Slc5a1, Slc9a3, Slc26a3, and Slc26a6 were all significantly decreased after Dox‐induced T‐bet expression (Figure 5a,b). Similar patterns were observed in Dox‐treated Tet‐O‐T‐bet:rtTA mice (Figure 5c,d), consistent with their sensitive phenotype after T‐bet overexpression. In contrast, the expression of these ion channels and transporters did not change in the WT control mice, with or without doxycycline treatment (Figure S7a,b, Supporting Information).

Further investigation was conducted using mouse intestinal organoids, which is a superior 3D in vitro model that better recapitulates tissue development and morphology, while retaining parental genomic and transcriptomic characteristics. After treated with different doses of doxycycline, T‐bet overexpression was clearly observed at 1 µg mL^−1^ dosage in the organoids from both Tet‐O‐T‐bet:rtTA and Tet‐O‐T‐bet:rtTA‐GFP:Villin‐Cre mice (Figure S7c, Supporting Information). We thus selected 1 µg mL^−1^ as the working dose, and observed significantly increased Cftr and Ano1, but decreased Slc5a1, Slc9a3, Slc26a3, and Slc26a6 in Dox‐treated Tet‐O‐T‐bet:rtTA‐GFP:Villin‐Cre organoids (Figure S7e, Supporting Information), consistent with their expression levels in the mouse intestine (Figure 5a). Similar patterns were observed in Dox‐treated Tet‐O‐T‐bet:rtTA organoids (Figure S7f, Supporting Information), although statistically significant changes only occurred in some of the candidates, probably because the T‐bet expression level in Tet‐O‐T‐bet:rtTA organoids was not as high as it was in Tet‐O‐T‐bet:rtTA‐GFP:Villin‐Cre organoids (Figure S7c, Supporting Information). As a negative control group, the WT organoids did not show any dramatic changes in these ion channels and transporters after doxycycline treatment (Figure S7d, Supporting Information). Overall, the data from tissue organoid system confirmed our observations in the in vivo studies.

Next, we aimed to investigate whether the therapeutic intervention of mouse diarrhea may ameliorate dehydration and survival in T‐bet overexpressed mice. Tet‐O‐T‐bet:rtTA‐GFP:Villin‐Cre mice were orally treated with an anti‐diarrhea drug Imodium (Loperamide hydrochloride), then treated with doxycycline water solution for T‐bet induction. We found that the anti‐diarrhea drug could dramatically improve the health condition of Dox‐treated mice, with a significantly ameliorated body weight loss (Figure 5e). Consistently, the mouse survival was significantly extended in the Imodium‐treated group, with 80% of mice surviving on day 8 after doxycycline treatment (Figure 5f). Taken together, these data suggest that T‐bet overexpression in gut epithelial cells alters the expression of ion channels and transporters and induces fatal diarrhea and dehydration, while the treatment of anti‐diarrhea drugs could ameliorate the symptoms.

### 2.6 Inducible T‐bet Expression Induces the Apoptosis of Intestinal Epithelial Cells

**Figure 6:**
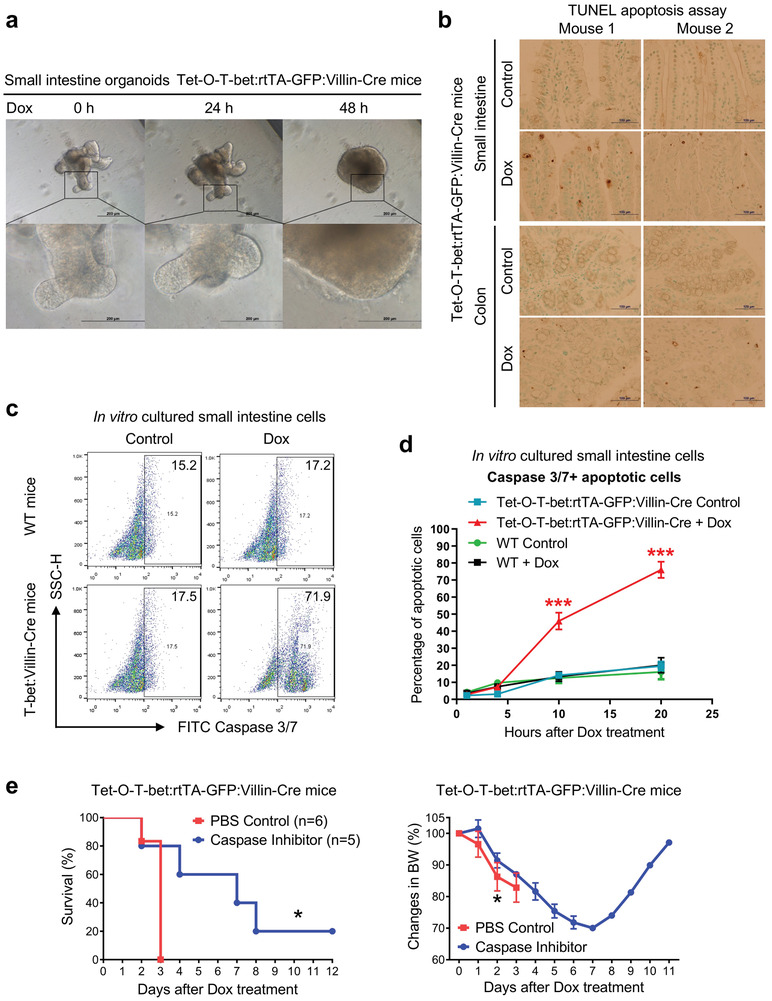
Inducible T‐bet expression promotes the apoptosis of intestinal epithelial cells. a) Small intestines were dissected from Tet‐O‐T‐bet:rtTA‐GFP:Villin‐Cre mice (gut epithelia specific T‐bet overexpression), and the organoids were cultivated and treated with 1 µg mL^−1^ doxycycline. The morphological changes of these organoids were observed at different time points under the microscope (scale bar: 1 mm for low magnification and 200 µm for high magnification). b) Tet‐O‐T‐bet:rtTA‐GFP:Villin‐Cre mice were treated with control water (5% sucrose alone) or doxycycline water solution (1 mg mL^−1^ in 5% sucrose) for 2 days. Mice were euthanized on day 2 for tissue collection. TUNEL staining was performed on intestinal sections to indicate the apoptotic cells after the induction of T‐bet overexpression (scale bar: 200 µm). c,d) Small intestines were dissected from WT and Tet‐O‐T‐bet:rtTA‐GFP:Villin‐Cre mice, washed in PBS and cut into small pieces, and in vitro treated with 1 µg mL^−1^ doxycycline in RPMI 1640 medium (supplemented with 10% FBS and penicillin–streptomycin). At different time points (0, 4, 10, 20 hours), tissue samples were digested, and the apoptotic cells were detected by flow cytometry after the staining of Caspase 3/7. c) Representative flow cytometry data at hour 20 to show the massive apoptotic cells in Dox‐treated Tet‐O‐T‐bet:rtTA‐GFP:Villin‐Cre intestines. The percentages of Caspase 3/7^+^ cells before and after doxycycline treatment were 15.2% and 17.2% in WT mice, and 17.5% and 71.9% in T‐bet overexpressed mice, respectively. d) Percentages of apoptotic cells in different groups after the doxycycline treatment (mean ± SD). e) Tet‐O‐T‐bet:rtTA‐GFP:Villin‐Cre mice were intraperitoneally (i.p.) injected with PBS control or pan‐Caspase inhibitor (Z‐VAD‐FMK, Sigma‐Aldrich, 50 µg in 200 µL PBS) every day from days 0 to 3. Doxycycline water solution was treated from days 0 to 7. The body weight changes (mean ± SD) and survival curves were observed and analyzed (*n* = 6 for control, *n* = 5 for experimental group). Statistical analyses: d,e,right) ANOVA and student's unpaired t test and e,left) Mantel‐Cox log‐rank test. *p < 0.05; ***p < 0.001.

The intestinal epithelium is the most highly regenerative tissue in the human body and is self‐renewed every 4–7 days from the Lgr5^+^ stem cells at the crypt base,^[^ ^20^ ^]^ allowing it to recover from constant damage during food breakdown, nutrient absorption, and waste elimination. The apoptosis of epithelial cells is a frequent event in the intestine and is essential for maintaining cellular balance.^[^ ^21^ ^]^ Under pathological conditions, such as inflammation and infection, an aberrant increase in apoptotic cells in the gut is commonly observed, accompanied by extensive epithelial erosion.^[^ ^22^ ^]^ Recent studies show that ion channels and transporters play a critical role in the apoptosis.^[^ ^23^ ^]^ Therefore, we reasoned that T‐bet may induce intestinal epithelial cell apoptosis. Indeed, when treating the Tet‐O‐T‐bet:rtTA‐GFP:Villin‐Cre organoids with doxycycline, we surprisingly found that the cells were mostly dying after 2 days, and the 3D structure of the organoids was severely damaged after the induction of T‐bet expression (**Figure** **6**a; Figure S8a, Supporting Information). Similar results were observed in Dox‐treated Tet‐O‐T‐bet:rtTA organoids, but not WT organoids (Figure S8b, Supporting Information), indicating that T‐bet overexpression not only induces animal mortality but also triggers the death of gut epithelial cells in the in vitro model. We performed a terminal deoxynucleotidyl transferase dUTP nick end labeling (TUNEL) assay on intestinal sections to detect the apoptotic cells. Indeed, we observed an increased amount of dark‐staining apoptotic cells in both small intestines and colons of T‐bet overexpressed mice (Figure 6b). However, the number of detected apoptotic cells is still too small for such a severe phenotype. We hypothesize that the apoptotic cells may have detached from the intestinal epithelia before sample collection, thus cannot be detected in tissue sections. To overcome this technical issue, we cultured the small intestine pieces in vitro, added doxycycline to the medium for T‐bet induction, and harvested all the cells in the plates. We found the caspase 3/7^+^ apoptotic cells were dramatically increased in Dox‐treated intestinal tissues from Tet‐O‐T‐bet:rtTA‐GFP:Villin‐Cre mice, but not WT control mice (Figure 6c). The percentages of apoptotic cells at different time points were statistically analyzed, and we observed massive cell apoptosis in over 70% of intestinal epithelial cells in 2 days after inducible T‐bet overexpression (Figure 6d).

To determine whether blockage of cell apoptosis may rescue the mortality of T‐bet‐overexpressing mice, we injected a pan‐caspase inhibitor Z‐VAD‐FMK into Dox‐treated Tet‐O‐T‐bet:rtTA‐GFP:Villin‐Cre mice. We found that the caspase inhibitor‐treated mice had significantly extended survival and ameliorated body weight loss (Figure 6e). About 20% of the mice survived beyond 12 days, with fully recovered body weights, after the stop of Dox treatment on day 7 (Figure 6e). These data suggest that T‐bet inducible expression directly triggers the apoptosis of intestinal epithelial cells, and the intervention of apoptotic pathways could partially but significantly protect mice from T‐bet‐induced mortality.

### 2.7 Inducible T‐bet Expression Inhibits Colon Cancer Development

**Figure 7:**
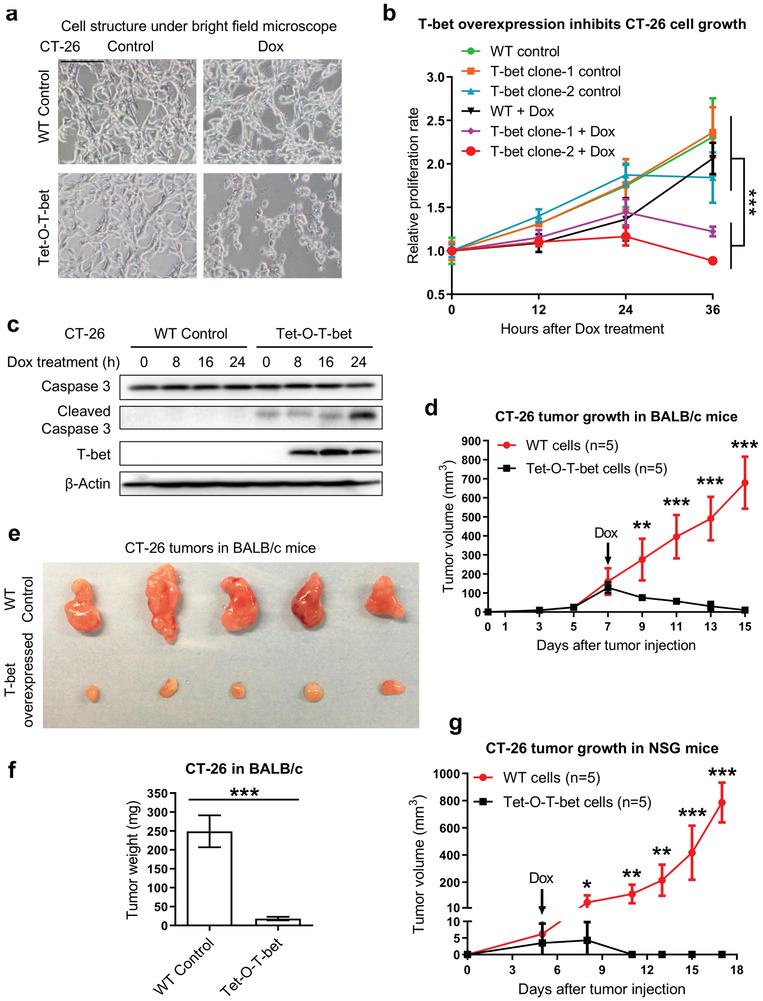
Inducible T‐bet expression inhibits colon cancer development. a) WT control and Tet‐O‐T‐bet transduced CT‐26 cells (clone #2) were treated with PBS control or 1 µg mL^−1^ doxycycline for 48 hours. The morphological change in cells with Dox‐induced T‐bet overexpression was observed under the microscope (scale bar: 200 µm). b) WT control and Tet‐O‐T‐bet transduced CT‐26 single clones were treated with PBS control or 1 µg mL^−1^ doxycycline. MTT assay was performed at different time points to determine the in vitro cell growth rates. Data were read at OD 590 nm and normalized with the value on hour 0 for the relative fold changes (mean ± SD). c) WT control and Tet‐O‐T‐bet transduced CT‐26 cells (clone #2) were treated with PBS control or 1 µg mL^−1^ doxycycline. Protein samples were harvested at different time points, and western blotting was performed to determine the activation of Caspase 3 in T‐bet overexpressed cells. d–f) WT control and Tet‐O‐T‐bet transduced CT‐26 cells were subcutaneously (s.c.) injected into WT BALB/c mice (*n* = 5), and continuously treated with doxycycline water solution (1 mg mL^−1^ in 5% sucrose) since day 7. d) Tumor sizes were measured every 2 days for the in vivo growth curves (mean ± SD). e) The image of tumors dissected on day 15 to compare the tumor sizes between two groups. f) Statistical analysis of the tumor weights (mean ± SD). g) WT control and Tet‐O‐T‐bet transduced CT‐26 cells were subcutaneously (s.c.) injected into NSG mice (*n* = 5), and continuously treated with doxycycline water solution since day 5. Tumor sizes were measured every 3 days for the in vivo growth curves (mean ± SD). Statistical analyses: f) Student's unpaired t test, b,d,g) ANOVA and student's unpaired t test. *p < 0.05; **p < 0.01; ***p < 0.001.

Following the discovery that T‐bet promotes apoptosis in intestinal epithelial cells, we next explored its therapeutic potential to control the growth and progression of epithelial‐type cancers in the intestine, such as colon cancer. To this end, we generated Tet‐O‐T‐bet‐transduced CT‐26 cells, in which T‐bet overexpression could be induced by doxycycline treatment. Two clones were picked, and inducible T‐bet expression was validated by western blotting (Figure S9a, Supporting Information). After doxycycline treatment, we found that Tet‐O‐T‐bet CT‐26 cells died massively, with dramatic morphological changes (**Figure** **7**a), while WT cells and untreated control cells grew vigorously. From the MTT (3‐[4,5‐dimethylthiazol‐2‐yl]‐2,5 diphenyltetrazolium bromide) assay, we found comparable in vitro growth rates between untreated WT and Tet‐O‐T‐bet CT‐26 cells (Figure 7b). However, after doxycycline treatment, the growth rates of Tet‐O‐T‐bet CT‐26 clones significantly declined (Figure 7b). Consistently, we observed an enhanced activation of caspase 3 in Tet‐O‐T‐bet CT‐26 cells after 1 day of doxycycline treatment (Figure 7c), thus proving the induction of cell apoptosis.

Furthermore, we injected WT and Tet‐O‐T‐bet CT‐26 cells into BALB/c mice for in vivo tumor development and found the two groups had similar levels of tumor growth before doxycycline treatment (Figure 7d). After doxycycline treatment on day 7, we found that WT tumors continued to grow, while the sizes of T‐bet‐expressing tumors were decreased, with significantly smaller volumes than WT controls at all following time points (Figure 7d). On day 15, tumors were harvested for measurement, and T‐bet‐expressing tumors had significantly reduced sizes and weights compared to WT controls (Figure 7e,f; Figure S9b, Supporting Information). Similar experiments were also performed in NSG mice, and consistent results were observed (Figure 7g). Notably, the Tet‐O‐T‐bet CT‐26 tumors totally disappeared at the final time point in NSG mice (Figure 7g), so we only showed the growth curves in our figure.

Besides CT‐26, we also validated the role of T‐bet in another colon cancer model, MC‐38 cells. Consistent with the CT‐26 data, we found that inducible T‐bet expression triggered morphological changes and cell apoptosis in both Tet‐O‐T‐bet MC‐38 clones (Figure S9c, Supporting Information). The cells were harvested and stained for flow cytometry analysis, and we found the percentage of caspase 3/7^+^ cells was significantly increased in Dox‐treated Tet‐O‐T‐bet MC‐38 clones compared to WT cells (Figure S9d,e, Supporting Information). Notably, the percentage of apoptotic cells tended to be higher in MC‐38 clone #2, in which the T‐bet expression was higher after Dox treatment (Figure S9f, Supporting Information). Similar results were also observed in Tet‐O‐T‐bet CT‐26 cells, in which clone #2 had relatively higher T‐bet expression and lower cell growth rate than clone #1 (Figure 7b; Figure S9a, Supporting Information). Altogether, these data suggest that the ectopic expression of T‐bet in colon cancer cells could induce apoptosis and tumor shrinkage, thus may be utilized as a novel approach for treating the patients with established tumors.

## 3 Discussion

In this study, we dissect the novel function of ectopic T‐bet expression in intestinal epithelial cells for the induction of local inflammation, diarrhea, apoptosis, and mouse mortality. T‐bet is a key transcription factor for T cell differentiation, and has long been implicated in the pathogenesis of IBD.^[^ ^8^, ^24^ ^]^ However, prior studies have only focused on immune cells but not intestinal epithelial cells, in which the role of T‐bet has never been reported. In our work, we unexpectedly found that inducible T‐bet expression in mice causes severe diarrhea, intestinal tissue damage, and mouse mortality. Instead of immune cells, T‐bet expression in intestinal epithelial cells is the critical determinant of the lethal phenotype. Mechanistically, we found that inducible T‐bet expression in gut epithelial cells alters the expression of ion channels and transporters, triggering massive fluid secretion and mouse dehydration. Furthermore, ectopic T‐bet expression promotes the apoptosis of gut epithelial cells. Thus, oncolytic viral delivery of T‐bet in tumor cells may be a promising approach to inhibiting the development and growth of colon and other cancers.

Although T‐bet is rarely investigated in non‐immune cells, other defining transcription factors for specific immune populations, such as GATA3 for Th2 cells and FOXP3 for Treg cells, have been widely reported as important regulators in epithelial cells. GATA3 is required for luminal epithelial cell differentiation in mammary gland,^[^ ^25^ ^]^ and also drives the transition from pluripotency to differentiation in human embryonic stem cells.^[^ ^26^ ^]^ FOXP3 serves as a tumor suppressor in breast cancer cells^[^ ^27^ ^]^ and inhibits the tumor metastasis,^[^ ^28^ ^]^ however, in non‐small cell lung cancer, FOXP3 promotes tumor growth and metastasis by activating Wnt/β‐catenin signaling pathway and epithelial–mesenchymal transition.^[^ ^29^ ^]^

The functions of T‐bet in colitis, inflammatory diseases, and cancer have been widely established; however, almost all previous studies have focused on its functions in immune cells. The roles of T‐bet in other cell and tissue types, such as gut epithelia in physiological conditions, are rarely reported. In fact, from our bioinformatic analyses in Figure S1 (Supporting Information), the expression levels of T‐bet in the digestive tissues (colon, small intestine, and stomach) are higher than the median expression level of all 147 tested healthy human tissues, even higher than some immune cell types such as Treg cells. Furthermore, T‐bet expression in intestinal epithelia is significantly up‐regulated in human patients with gut inflammation‐related diseases (enteritis of small intestine, Crohn's disease, and ulcerative colitis),^[^ ^10^, ^30^ ^]^ as well as in the mouse inducible colitis model.^[^ ^11b^ ^]^ In this study, we identified a novel mechanism of T‐bet in gut epithelial cells that can possibly explain these clinical observations. By using a tissue‐specific T‐bet inducible expression system, we found that the overexpression of T‐bet in epithelial cells could (1) trigger liquid secretion by regulating the ion channels and transporters, and (2) induce cell apoptosis. Therefore, enhanced T‐bet expression in human patients will promote the diarrhea, dehydration, and intestinal tissue damage, thus is relevant to the disease progression.

Ion channels and transporters have been widely reported to control diarrhea and dehydration. Among all the tested candidates, CFTR and ANO1 are the most important chloride channels that promote water secretion.^[^ ^18^ ^]^ The CFTR gene is strongly expressed all along the intestinal tract,^[^ ^31^ ^]^ and its prolonged activation leads to excessive outward movement of water and the anions Cl^−^ and HCO3^−^ into the lumen, resulting in secretory diarrhea.^[^ ^32^ ^]^ Mutations of CFTR affect the chloride ion channel function and lead to dysregulation of epithelial fluid transport in the lung, pancreas, and other organs, resulting in cystic fibrosis.^[^ ^33^ ^]^ The other chloride channel, ANO1, also plays a key role in fluid and electrolyte secretion. Its abnormal expression is involved in the pathology of various diseases, including asthma, diarrhea, cystic fibrosis, hypertension, ischemic stroke, and cancer.^[^ ^34^ ^]^ As a result, both CFTR and ANO1 are considered emerging therapeutic targets for diarrhea and other epithelium‐originated diseases, and some inhibitors are currently under clinical trials.^[^ ^34^, ^35^ ^]^ In our study, we observed increased levels of Cftr and Ano1 in mouse small intestine, colon, and gut organoids after T‐bet overexpression, consistent with the diarrhea and dehydration phenotypes.

At the same time, SLC family members are key ion transporters that mediate the uptake of chloride and sodium ions, thus promoting water absorption.^[^ ^18a^ ^]^ Compared with normal human colons, the mRNA levels of SLC26A3 and SLC9A3 decrease, while CFTR increases, in ulcerative colitis patients.^[^ ^36^ ^]^ In the DSS colitis model, deletion of Slc26a3 results in dramatically reduced fluid absorption, loss of the adherent mucus layer, and severely increased colonic mucosal damage.^[^ ^37^ ^]^ In human patients, reduced SLC9A3 activity causes congenital sodium diarrhea.^[^ ^38^ ^]^ SLC26A6 not only participates in ion homeostasis and acid‐base balance to control intestinal diseases, but also serves a significant role in mediating nephrolithiasis, fetal skeletal dysplasia, arrhythmia, and pancreatic diseases.^[^ ^19^ ^]^ In our study, we observed decreased Slc5a1, Slc9a3, Slc26a3, and Slc26a6 in T‐bet overexpressed intestinal epithelial cells, consistent with the mouse phenotype. The detailed molecular mechanism of how T‐bet regulates the expression of these ion channels and transporters merits future investigation.

Severe diarrhea leads to dehydration and electrolyte loss, thus is a key reason for animal death we observed in T‐bet overexpressed mice. These symptoms are similar to the human secretory diarrhea, which is predominantly due to the robust secretion of chloride and bicarbonate ions.^[^ ^39^ ^]^ One prototypical example of secretory diarrhea is cholera,^[^ ^40^ ^]^ in which *Vibrio cholerae* produces the cholera toxin and triggers the activation of CFTR.^[^ ^41^ ^]^ Without medical treatment, secretory diarrhea can lead to severe electrolyte abnormalities, acidosis, acute renal failure, hypovolemic shock, and death in human patients. To rescue the T‐bet overexpressed mice and extend their survival, we treated the mice with an FDA‐approved anti‐diarrhea drug, Imodium (loperamide hydrochloride). As a potent opioid receptor agonist, Imodium inhibits enteric nerve activity and neurotransmitter release, suppresses acetylcholine and prostaglandins to reduce propulsive peristalsis, and stimulates intestinal absorption of water and electrolytes by inhibiting calmodulin.^[^ ^42^ ^]^ We found that Imodium treatment could significantly extend the survival of T‐bet overexpressed mice, with 80% surviving at the endpoint. These data suggest that secretory diarrhea is a dominant, but probably not the only, reason for mouse mortality.

Cell apoptosis frequently occurs in intestinal epithelia. With constant damage during food digestion, the intestinal epithelium is self‐renewed every 4–7 days, thus becoming the most highly regenerative tissue in the human body.^[^ ^20a^ ^]^ Under pathological conditions, such as inflammation and infection, intestinal cell apoptosis dramatically increases, together with tissue damage.^[^ ^22^ ^]^ After the induction of T‐bet expression, we observed dramatically enhanced caspase 3/7^+^ apoptotic cells in the mouse intestine and structural damage in tissue organoids, confirming that T‐bet promotes apoptosis in gut epithelial cells. The increase in cleaved‐caspase 3 in T‐bet‐expressing colon cancer cells also supports this finding. Furthermore, the treatment of an anti‐apoptosis drug (pan‐caspase inhibitor Z‐VAD‐FMK) significantly extended the overall survival and rescued a small population of mice from death. Notably, in the CT‐26 and MC‐38 models, cell clones with higher T‐bet expression levels had lower in vitro growth rates and higher percentages of caspase 3/7^+^ cells, indicating that the severity of cell apoptosis is related to the expression level of T‐bet. Therefore, enhanced apoptosis is another potential reason for mouse mortality in T‐bet‐overexpressed mice. Although both anti‐diarrhea and anti‐apoptosis drugs can partially rescue T‐bet‐overexpressed mice, we did not perform a combination of the two drugs. Both secretory diarrhea and cell apoptosis have complex mechanisms and include numerous regulator factors. The simple combination of the two drugs will lead to an unpredictable result that is more complicated to explain; thus, it is not a rational design in our study. Collectively, our results suggest that T‐bet overexpression upregulates expression of ion channels and transporters, and induce apoptosis in gut epithelial cells.

Dox‐induced T‐bet expression triggered the apoptosis of cancer cells and led to in vivo tumor shrinkage. At later time points, the tumors were extremely small or even disappeared in the T‐bet‐expressing group. Therefore, the induction of ectopic T‐bet expression in cancer cells can be a novel strategy for effectively eliminating epithelial‐type cancers. In recent years, oncolytic virus therapy has been developed as a new form of immunotherapy, using viruses to infect and destroy cancer cells.^[^ ^43^ ^]^ Tumor‐specific infection of engineered oncolytic viruses can be utilized to enable T‐bet expression in cancer cells, but not normal cells, thus facilitating tumor clearance. Since most of the current immunotherapies do not show good responses in solid tumors, our finding has great clinical significance for the treatment of cancer patients.

## 4 Conclusion

In summary, our study delineates a previously unidentified function of T‐bet in dictating the fate of intestinal epithelial cells, through regulating the cell apoptosis and expression of ion channels and transporters. Although T‐bet overexpression in gut epithelial cells is lethal in the mouse model due to secretory diarrhea and tissue damage, it effectively triggers the shrinkage of developed tumors in xenograft models, thus implicating a promising therapeutic potential in future cancer treatment, particularly in the realm of epithelial‐derived cancers.

## 5 Experimental Section

### Animals and In Vivo Procedures

Tet‐O‐T‐bet mice were generated in the lab. Other strains were purchased from The Jackson Laboratory, including the wild‐type (WT) C57BL/6 control mice (Cat#: 000664), rtTA mice (whole body, Cat#: 006965), rtTA‐EGFP mice (tissue specific, Cat#: 005572), Lyz2‐Cre mice (Cat#: 004781), CD4‐Cre mice (Cat#: 017336), and Villin‐Cre mice (Cat#: 004586). Mice were crossed in the lab to generate different strains for experiments, including: Tet‐O‐T‐bet:rtTA mice (systemic T‐bet inducible expression), Tet‐O‐T‐bet:rtTA‐EGFP:CD4‐Cre mice (CD4 T cell specific T‐bet inducible expression), Tet‐O‐T‐bet:rtTA‐EGFP:Lyz2‐Cre mice (myeloid cell specific T‐bet inducible expression), and Tet‐O‐T‐bet:rtTA‐EGFP:Villin‐Cre mice (gut epithelia specific T‐bet inducible expression). For tumor studies, WT BALB/c mice (Cat#: 000651) and NSG mice (Cat#: 005557) were ordered from The Jackson Laboratory. In all experiments, 6 to 8 weeks old mice were used unless specifically described. All the mice were housed under a 12:12 light:dark cycle, and maintained in specific pathogen‐free facilities that are accredited by Association for Assessment and Accreditation of Laboratory Animal Care International (AAALAC). All animal studies were approved by the Institutional Animal Care and Use Committee (IACUC) of Houston Methodist Research Institute (#IS00004400) and University of Southern California (#21097).

Tet‐O‐T‐bet transgenic mouse strain was generated by incorporating the mouse *Tbx21* coding sequence into a doxycycline‐inducible expression system (Figure S2a, Supporting Information). To construct the tet response element (TRE), 7 repeats of tetracycline operator sequence (7× tet) were fused to a minimal cytomegalovirus (CMV) promoter (mCMV), which lacks an enhancer sequence. The constructed DNA fragments, containing TRE‐Tbx21 and homologous arm, were linearized, purified, and microscopically injected into the fertilized eggs and transferred into the pseudo‐pregnant female mice for generating the Tet‐O‐T‐bet transgenic pups. The expression of reverse tetracycline‐controlled transactivator (rtTA) from either rtTA or rtTA‐EGFP mouse strains was controlled by a constitutive or tissue‐specific promoter, respectively. Without doxycycline (Dox), rtTA exhibits a reverse phenotype and cannot bind to the Tet‐On sequences. In the presence of doxycycline, rtTA binds to the Tet‐On sequences and recruits RNA polymerase II factors for the assembly of transcription complexes, initiating the transcription of T‐bet gene.

In mouse survival assay, mice were continuously treated with control water (5% sucrose alone) or doxycycline water solution (1 mg mL^−1^ in 5% sucrose) through the drinking water. Body weight of each mouse was measured daily, and the survival was observed until the sensitive groups were all euthanized, while the resistant groups remained healthy.

To generate bone marrow chimeric mice, recipient WT and Tet‐O‐T‐bet:rtTA mice were γ‐irradiated at 1000 rad (or 10 Gy), and intravenously injected with 5 million fresh bone marrow cells from donor mice after 24 hours. Recipient mice were treated with antibiotic water (0.5 mg mL^−1^ Baytril, Bayer) for 2 weeks starting from 2 days before irradiation. Regular drinking water was replaced to the mice after antibiotic water treatment. Thirty‐five days after the bone marrow transfer, chimeric mice were treated with doxycycline water solution (1 mg mL^−1^ in 5% sucrose) for future experiments.

For immune cell depletion, Tet‐O‐T‐bet:rtTA mice were intraperitoneally (i.p.) injected with clodronate liposomes (150 µL per injection, C‐020, Liposoma BV) on days ‐2/0/+2, and a combination of Ly‐6G antibody (Ab) (250 µg per injection, BE0075‐1, BioXCell), CD4 Ab (300 µg per injection, TIB‐207, ATCC), and CD8 Ab (300 µg per injection, TIB‐210, ATCC) on days ‐1/+1/+3, to simultaneously deplete macrophages, neutrophils, and T cells. PBS‐control liposomes and isotype‐control Abs were used in the control group. For each injection, 200 µL volume was used (supplemented with PBS). On day 0, blood samples were collected to validate the depletion efficacy by flow cytometry. Mice were continuously treated with doxycycline water solution (1 mg mL^−1^ in 5% sucrose) from day 0. The body weights and survival curves were observed.

For testing the changes in the ion channels and transporters, different groups of mice were treated with control water (5% sucrose alone) or doxycycline water solution (1 mg mL^−1^ in 5% sucrose) for 2 days. Mice were euthanized on day 2, and RNA samples were isolated from PBS‐cleaned small intestine and colon tissues for real‐time QPCR.

For anti‐diarrhea drug treatment, Tet‐O‐T‐bet:rtTA‐GFP:Villin‐Cre mice were orally treated with PBS control or Imodium (125 µg Loperamide hydrochloride in 200 µL PBS) every day since day ‐1. Drinking water with doxycycline (1 mg mL^−1^ in 5% sucrose) was continuously treated since day 0. The body weights and survival curves were observed.

For anti‐apoptosis drug treatment, Tet‐O‐T‐bet:rtTA‐GFP:Villin‐Cre mice were intraperitoneally (i.p.) injected with PBS control or pan‐Caspase inhibitor (Z‐VAD‐FMK, Sigma‐Aldrich, 50 µg in 200 µL PBS) every day from days 0 to 3. Doxycycline water solution (1 mg mL^−1^ in 5% sucrose) was treated from days 0 to 7. The body weights and survival curves were observed and analyzed.

For in vivo tumor studies, WT and inducible T‐bet expressing CT‐26 cells (0.5 million cells in 50 µL PBS + 50 µL Matrigel) were subcutaneously (s.c.) injected into WT BALB/c or NSG mice. Doxycycline water solution (1 mg mL^−1^ in 5% sucrose) were continuously treated since day 7 in BALB/c mice, or day 5 in NSG mice. Tumor sizes were observed every 2–3 days. For BALB/c mice, tumors were dissected on day 15 to compare the tumor sizes between two groups. For NSG mice, the tumors in T‐bet overexpression group disappeared after day 11, therefore, only the growth curves were showed.

### Histology and Tissues Staining

Fresh organs and tissues were fixed with 3.7% formalin for 24 hours, and then sent to the histology laboratory at Baylor Breast Care Center or USC School of Pharmacy for further processing and H&E staining. Images were acquired using the Olympus BX61 microscope along with DP71 digital camera (Olympus).

IHC staining was performed following previously published protocol.^[^ ^44^ ^]^ Briefly, unstained tissue sections were deparaffinized in xylene, rehydrated in graded ethanol solutions, and washed in tap water. Antigen retrieval was achieved by boiling the slides in a pressure cooker for 3 min in a citrated buffer (10 × 10^−3^ m trisodium citrate, pH 6.0). After 10 min treatment with 3% H_2_O_2_, tissue sections were blocked with 5% normal goat serum in TBST for 1 hour at room temperature, incubated with primary antibodies at 4 °C overnight, and with EnVision Polymer‐HRP secondary antibodies (Dako) at room temperature for 30 min. After the application of DAB chromogen (Vector), tissue sections were stained with hematoxylin, dehydrated, and mounted. The antibodies used for IHC staining are listed in Table S1, Supporting Information.

TUNEL staining was performed on intestinal sections to indicate the apoptotic cells, using the TumorTACS In Situ Apoptosis Detection Kit (Cat#: 4815‐30‐K, R&D Systems), following the manufacturers’ instructions.

### Western Blotting

For both mouse tissues and cultured cells, RIPA buffer (150 × 10^−3^ m NaCl, 50 × 10^−3^ m Tris‐HCl pH 7.4, 2 × 10^−3^ m EDTA, 0.1% SDS, and 1% NP‐40; supplemented with Roche protease inhibitor cocktail) was used for cell lysis and protein extraction. Tissues were homogenized in RIPA buffer on ice for thorough lysis. After shaking the samples at 4 °C for 30 min, tissue/cell debris was removed by centrifugation for 3 min at 12 000 *g* at 4 °C in a microcentrifuge. Lysate was mixed with 5X SDS loading buffer and heated for 5 min at 100 °C. Prepared samples were resolved by 10% SDS‐PAGE and transferred to PVDF membranes (Bio‐Rad). The membrane was blocked for 1 hour at room temperature using blocking buffer (5% nonfat dried milk in TBST). Blots were incubated with appropriate primary antibodies (diluted at 1:1000) and HRP‐conjugated secondary antibodies (diluted at 1:3000). Protein expression was normalized to β‐actin antibody (diluted at 1:3000). HRP was detected using chemiluminescent HRP substrate (Millipore). Digital images were acquired with the ChemiDoc XRS+ System and analyzed by Image Lab v.5.1 (Bio‐Rad). The antibodies for Western blotting are listed in Table S1, Supporting Information.

### Flow Cytometry and Sorting

For surface marker staining, cells were directly stained in a cocktail of fluorochrome‐conjugated antibodies for 30 min on ice, washed twice in 2% FBS/PBS, and resuspended in 2% FBS/PBS for flow cytometry. For intracellular staining, cells were activated with PMA (phorbol‐12‐myristate 13‐acetate), Ionomycin, and protein transport inhibitor for 4 hours at 37 °C. Then the cells were resuspended in Fix/Perm buffer (BD) and treated for 20 min on ice, washed with Perm/Wash buffer (BD), and stained with the antibody cocktail for 30 min on ice. After washing twice, cells were resuspended in 2% FBS/PBS for flow cytometry. BD LSR II analyzer was used to distinguish different subsets. For the sorting of T and B cells, mouse splenocytes were purified from control water or doxycycline water solution treated Tet‐O‐T‐bet:rtTA‐GFP:CD4‐Cre mice on day 2. Cells were stained with fluorochrome‐conjugated antibodies for 10 min at room temperature. After washing twice with 2% FBS/RPMI‐1640, cells were filtered with 40 µm strainer, and resuspended in 2% FBS/RPMI‐1640 for sorting. BD FACSAria II sorter was used to acquire the CD45^+^/B220^+^/CD3^‐^ B cells and CD45^+^/B220^‐^/CD3^+^ T cells. The antibodies for flow cytometry are listed in Table S1, Supporting Information.

### Mouse Genotyping

For genotyping, 0.5 cm length of mouse tail tip were collected for DNA purification. The tail tips were digested in 0.1 ml DNA digestion buffer (Viagen, #102‐T) with Proteinase K (0.5 mg mL^−1^ final concentration), and incubated overnight at 56 °C. The lysates were heated at 100 °C for 3 min to inactivate the Proteinase K, then directly used for genotyping PCR. Red Master Mix (2×, VWR) was used for genotyping PCR. The samples were run at “95 °C 30 seconds, 57 °C 30 seconds, 72 °C 1 minute” for 35 cycles. The primers for genotyping PCR are listed in Table S2, Supporting Information.

### Real‐Time QPCR Analysis

Total RNA was isolated from mouse tissues or cultured cells by TRIzol Reagent (Invitrogen), and the first‐strand cDNA was generated from total RNA using SuperScript IV Reverse Transcriptase (Thermo Fisher Scientific), following the manufacturers’ instructions. Real‐time qPCR was performed using iTaq Universal SYBR Green Supermix (Bio‐Rad) and specific primers on the Applied Biosystems QuantStudio 6 Flex Real‐Time PCR system (Thermo Fisher Scientific). The results were analyzed using QuantStudio Software v.1.3 (Thermo Fisher Scientific). Relative expression of target genes was normalized with mouse *Gapdh* primers. The primers for real‐time qPCR are listed in Table S2, Supporting Information.

### Small Intestine Organoid Culture

Using previously reported method,^[^ ^45^ ^]^ small intestinal crypts were purified from untreated mice in different genotypes for the 3D culture of organoids. Briefly, small intestines were dissected, opened longitudinally, and cleaned with cold PBS. Villi were scraped with a hemacytometer coverslip, leaving only the crypts. The intestine tissues were cut into 5 mm pieces, washed with cold PBS for 10–20 times, and incubated in 2 × 10^−3^ m EDTA in PBS for 30 min on ice. After the removal of EDTA solution, gut pieces were vigorously suspended in 10% FBS/PBS with a 10 mL pipette, and the supernatant was collected as one fraction. The wash steps were repeated, and more fractions were collected. Each fraction was filtered with a 70 µm strainer, centrifuged and resuspended for enrichment, and examined under microscope for the number and purity of crypts. Proper fractions consisting of essentially pure crypts were used for culture. Crypts were mixed with cold Matrigel (Cat#: 356234, Corning) and seeded in pre‐cooled 24‐well plates (100‐1000 crypts in 50 µL Matrigel per well). After 5–10 minutes’ incubation at 37 °C, 500 µL IntestiCult mouse organoid growth medium (Cat#: 06005, Stemcell) was added into each well. The organoids were maintained at 37 °C in a humidified atmosphere with 5% CO_2_. The culture medium was replaced every 2–3 days.

### Generation of Tet‐O‐T‐bet Cancer Cells

To generate doxycycline‐inducible expression of T‐bet (Tet‐O‐T‐bet) in CT‐26 and MC‐38 cell lines, two types of lentiviral particles were produced by transfecting HEK293T cells with pl‐TRE‐T‐bet, VSV‐G, and ∆8.9; or pl‐CMV‐rtTA, VSV‐G, and ∆8.9. The lentiviruses were further concentrated by ultracentrifugation. Colon cancer cells were transduced by incubating with mixed lentivirus for 24 hours and then replaced with fresh medium. Three days after transduction, the cells were treated with doxycycline (1 µg mL^−1^) for 24 hours, and T‐bet expression was verified by western blotting. T‐bet‐positive polyclonal cells were subsequently seeded into 96‐well plates for monoclonal selection. T‐bet‐positive monoclonal cells were picked and identified by western blotting. Monoclonal cells expressing high levels of T‐bet were selected for further functional studies.

### Cell Viability Assay

Using previously reported technique,^[^ ^46^ ^]^ the MTT assay was used to determine the in vitro growth rate of cancer cells with inducible T‐bet expression. Briefly, cells were cultured in 96‐well plates at 1000 cells per well and incubated at 37 °C with 5% CO_2_. MTT solution was prepared in PBS at 5 mg mL^−1^. PBS control or 1 µg mL^−1^ doxycycline was added into the cells at hour 0. At each time points (12, 24, 36 hours), 20 µL MTT solution was added into the 200 µL cell culture medium. After 4 hour incubation at 37 °C, the medium was discarded and 100 µL DMSO was added into each well. The 96‐well plates were then covered with aluminum foil and shaken for 10 min (360 rpm) at room temperature. The absorbance was read at OD = 590 nm on Biotek Synergy 2 microplate reader. Data were normalized with the value on hour 0 for the relative fold changes.

### Statistical Analysis

Descriptive statistics, including means, standard deviations, medians, and ranges, were computed for each group and analyzed with Student's t‐test or for multiple comparisons, with ANOVA. Data are presented as mean ± standard deviation (SD) in all the figures, as also described in figure legends. Differences in mice survival were evaluated with Mantel‐Cox log‐rank test. The sample size (*n*) for each experiment is included in the figure legends. All analyses were performed with GraphPad Prism 5 (GraphPad Software, La Jolla, CA). *P*‐values < 0.05 were considered significant.

## Conflict of Interest

The authors declare no conflict of interest.

## Author Contributions

L.C., H.Y., C.X., and Q.L. contributed equally to this work. R.‐F.W. and C.X. supervised the entire project. R.‐F.W., C.X., H.Y., Q.L., and L.C. designed the experiments. L.C., H.Y., C.X., and Q.L. performed the experiments and collected the data. T.D., X.L., and L.L. provided assistances in some experiments. L.C., H.Y., C.X., Q.L., and H.Y.W. performed the data analyses. C.X., H.Y., L.C., and R.‐F.W. wrote the paper, with input from others.

## Supporting information

Supporting Information

## Data Availability

The data that support the findings of this study are available from the corresponding author upon reasonable request.
