# Supplementary material for: T‐bet Regulates Ion Channels and Transporters and Induces Apoptosis in Intestinal Epithelial Cells
Source: Adv Sci (Weinh). 2024 Apr 22;11(28):2401654. doi: 10.1002/advs.202401654 (PMC11267362; doi:10.1002/advs.202401654)
Supplement: Supplementary file 1 — Supporting Information [file ADVS-11-2401654-s001.docx]

Supporting Information

**T-bet Regulates Ion Channels and Transporters and Induces Apoptosis in Intestinal Epithelial Cells**

*Lang Chen^1,2,#^, Hongwei Yi^1,3,#^, Qingtian Li^1,4,#^, Tianhao Duan^1,5^, Xin Liu^1,5^, Linfeng Li^1,6^, Helen Y. Wang^1,5^, Changsheng Xing^1,5,#,*^, and Rong-Fu Wang^1,5,7,8,*^*

^1^ Center for Inflammation and Epigenetics, Houston Methodist Research Institute, Houston, TX 77030, USA

^2^ Department of General Surgery, Third Xiangya Hospital, Xiangya School of Medicine, Central South University, Changsha 410013, China

^3^ Department of Pharmacology, School of Medicine, Southeast University, Nanjing 210009, China

^4^ Department of Medicine, Baylor College of Medicine, Houston, TX 77030, USA

^5^ Department of Medicine, Keck School of Medicine, University of Southern California, Los Angeles, CA 90033, USA

^6^ Department of Thoracic Surgery, Xiangya Hospital, Central South University, Changsha 410008, China

^7^ Department of Pediatrics, Children’s Hospital Los Angeles, Keck School of Medicine, University of Southern California, Los Angeles, CA 90027, USA

^8^ Norris Comprehensive Cancer Center, Keck School of Medicine, University of Southern California, Los Angeles, CA 90033, USA

^#^ These authors contributed equally to this work.

* Corresponding Author: Changsheng Xing, E-mail: [cxing999@usc.edu](mailto:cxing999@usc.edu); Rong-Fu Wang (Lead Contact), E-mail: [rongfuwa@usc.edu](mailto:rongfuwa@usc.edu).

**Table of Contents**

**Table S1.** Antibodies used in this study…………………………………………………….………..……3

**Table S2.** Primers used in this study………………………….……………………….….………………..4

**Figure S1.** TBX21 expression in normal human tissues……………………………….………………….5

**Figure S2.** Generation of Tet-O-T-bet:rtTA mice…………………………………………………………7

**Figure S3.** Tissue structures and T-bet inducible expression in doxycycline-treated Tet-O-T-bet:rtTA mice………………………………………………………………………………………………………..9

**Figure S4.** Inducible expression of T-bet in CD4^+^ T cells does not cause mouse mortality………….…11

**Figure S5.** Inducible expression of T-bet in myeloid cells does not cause mouse mortality…………….13

**Figure S6.** Inducible expression of T-bet in gut epithelial cells causes mouse mortality………………..15

**Figure S7.** T-bet regulates the expression of ion channels and transporters in mouse intestine…………17

**Figure S8.** Inducible T-bet expression promotes the apoptosis of intestinal epithelial cells…………….19

**Figure S9.** Inducible T-bet expression completely inhibits colon tumor formation and growth…………21

**Table S1. Antibodies used in this study**

| Antibody | Purpose | Source | Identifier |
| --- | --- | --- | --- |
| Anti-T-bet | IHC staining/Western blotting | Thermo Fisher | Cat#: 14-5825-82 |
| HRP-linked mouse 2^nd^ antibody | IHC staining | DAKO | Cat#: K4001 |
| Anti-Caspase 3 | Western blotting | Cell Signaling | Cat#: 9662S |
| Anti-Cleaved Caspase 3 | Western blotting | Cell Signaling | Cat#: 9661S |
| Anti-Flag | Western blotting | Sigma-Aldrich | Cat#: A8592 |
| Anti-β-Actin | Western blotting | Sigma-Aldrich | Cat#: A1978 |
| HRP-linked rabbit 2^nd^ antibody | Western blotting | Thermo Fisher | Cat#: 31460 |
| HRP-linked mouse 2^nd^ antibody | Western blotting | Thermo Fisher | Cat#: 31430 |
| Anti-T-bet | Flow cytometry | Thermo Fisher | Clone: eBio4B10 |
| Anti-F4/80 | Flow cytometry | Thermo Fisher | Clone: BM8 |
| Anti-CD11b | Flow cytometry | Thermo Fisher | Clone: M1/70 |
| Anti-Ly-6G | Flow cytometry | Thermo Fisher | Clone: RB6-8C5 |
| Anti-CD3 | Flow cytometry | Thermo Fisher | Clone: 17A2 |
| Anti-CD4 | Flow cytometry | Thermo Fisher | Clone: RM4-5 |
| Anti-CD8a | Flow cytometry | BD Biosciences | Clone: 53-6.7 (RUO) |
| Anti-CD49b | Flow cytometry | Thermo Fisher | Clone: DX5 |
| Anti-CD45 | Flow cytometry | Thermo Fisher | Clone: 30-F11 |
| Anti-IFN-γ | Flow cytometry | Thermo Fisher | Clone: XMG1.2 |
| Anti-B220 | Flow cytometry | Thermo Fisher | Clone: RA3-6B2 |
| Anti-CD4 | *In vivo* depletion | ATCC | Cat#: TIB-207 |
| Anti-CD8 | *In vivo* depletion | ATCC | Cat#: TIB-210 |
| Anti-Ly-6G | *In vivo* depletion | BioXCell | Cat#: BE0075-1 |

**Table S2. Primers used in this study**

| Primer | Purpose | Sequence (5’-3’) |
| --- | --- | --- |
| F: Tet-O-T-bet | Genotyping | GCGCGGCGATCGGTCGGGTC |
| R: Tet-O-T-bet | Genotyping | CAACTACATCCTGGTAATCATC |
| F: rtTA (Common) | Genotyping | AAAGTCGCTCTGAGTTGTTAT |
| R: rtTA (Mutant) | Genotyping | GCGAAGAGTTTGTCCTCAACC |
| R: rtTA (WT) | Genotyping | GGAGCGGGAGAAATGGATATG |
| F: EGFP | Genotyping | AGGGCGAGGAGCTGTTCA |
| R: EGFP | Genotyping | TGAAGTCGATGCCCTTCAG |
| F: CD4-Cre (Mutant) | Genotyping | GCGGTCTGGCAGTAAAAACTATC |
| R: CD4-Cre (Mutant) | Genotyping | GTGAAACAGCATTGCTGTCACTT |
| F: CD4-Cre (WT) | Genotyping | CTAGGCCACAGAATTGAAAGATCT |
| R: CD4-Cre (WT) | Genotyping | GTAGGTGGAAATTCTAGCATCATCC |
| F: Lyz2-Cre (Common) | Genotyping | CTTGGGCTGCCAGAATTTCTC |
| R: Lyz2-Cre (Mutant) | Genotyping | CCCAGAAATGCCAGATTACG |
| R: Lyz2-Cre (WT) | Genotyping | TTACAGTCGGCCAGGCTGAC |
| F: Villin-Cre | Genotyping | CATGTCCATCAGGTTCTTGC |
| R: Villin-Cre | Genotyping | TTCTCCTCTAGGCTCGTCCA |
| F: T-bet | Real-time qPCR | TTCTATCCAACCAGTATC |
| R: T-bet | Real-time qPCR | CTGTGAGATCATATCCTT |
| F: Ano1 | Real-time qPCR | AGGAGGATCATCCCAGAGCA |
| R: Ano1 | Real-time qPCR | GCTTCACCTTGTCGGTCTCT |
| F: Cftr | Real-time qPCR | GTTGATGGCAACCAGTCCGA |
| R: Cftr | Real-time qPCR | TCTGCCAGAGCAAGGTTGAA |
| F: Slc5a1 | Real-time qPCR | ATGCGGCTGACATCTCAGTC |
| R: Slc5a1 | Real-time qPCR | ACCAAGGCGTTCCATTCAAAG |
| F: Slc9a3 | Real-time qPCR | CACCCCTCCACGGCTAATAC |
| R: Slc9a3 | Real-time qPCR | TGAGTGACCCAGTCTGTTGT |
| F: Slc26a3 | Real-time qPCR | GCTTTAGCAGGTCCAGGGAA |
| R: Slc26a3 | Real-time qPCR | CGGATCTTTGGCCACCTACA |
| F: Slc26a6 | Real-time qPCR | TGCGGAGCCTTAGTTTACCG |
| R: Slc26a6 | Real-time qPCR | ACTCCTCGGTAAGCAGCAAC |
| F: Gapdh | Real-time qPCR | AGGTCGGTGTGAACGGATTTG |
| R: Gapdh | Real-time qPCR | TGTAGACCATGTAGTTGAGGTCA |

**
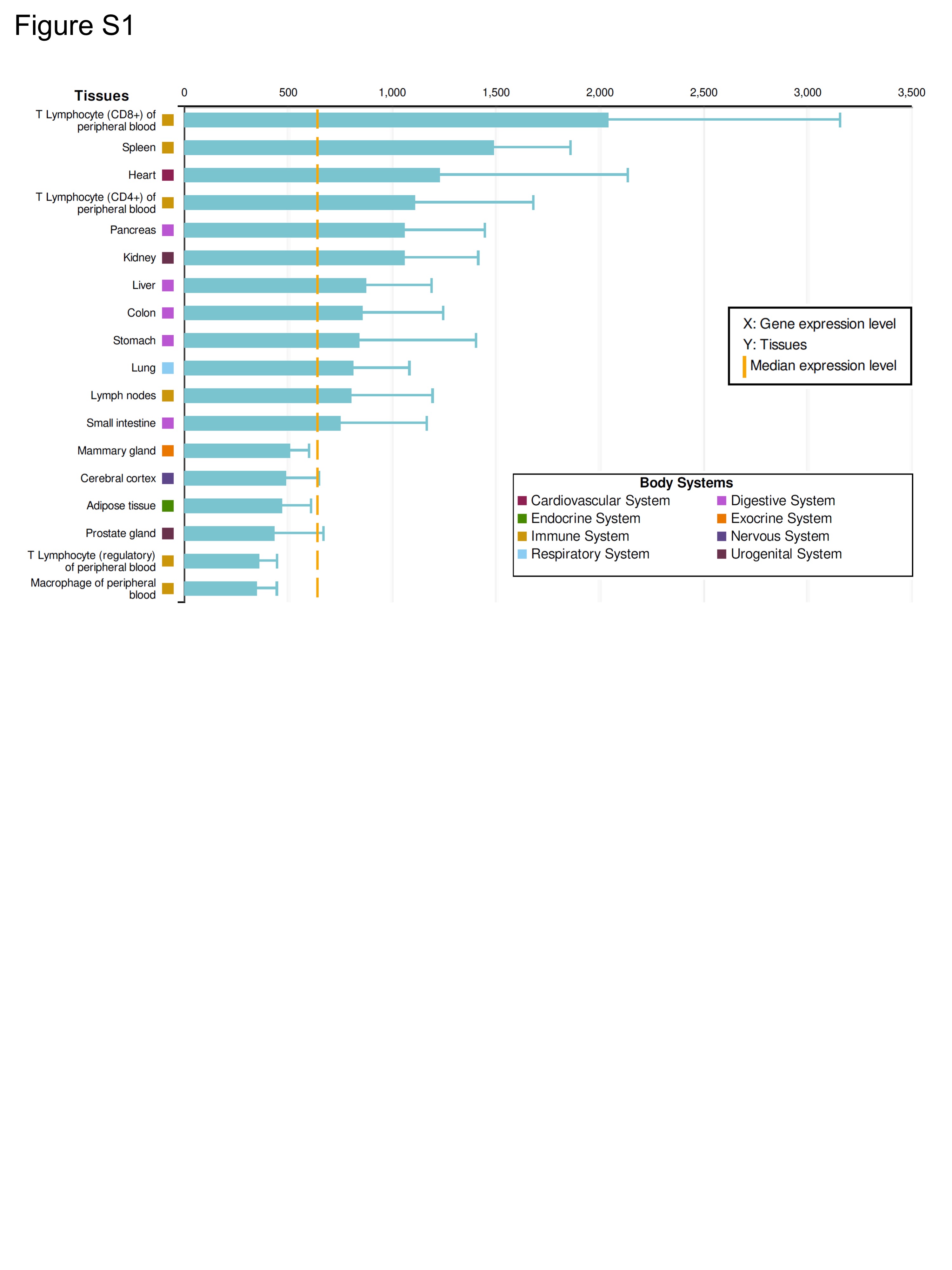
**

**Figure S1. TBX21 expression in normal human tissues.** Body Atlas analysis by BaseSpace Correlation Engine 2.0 from Illumina Inc. Selected major organs/tissues were included in the figure from all 147 tested human tissue types (mean ± SD).

**
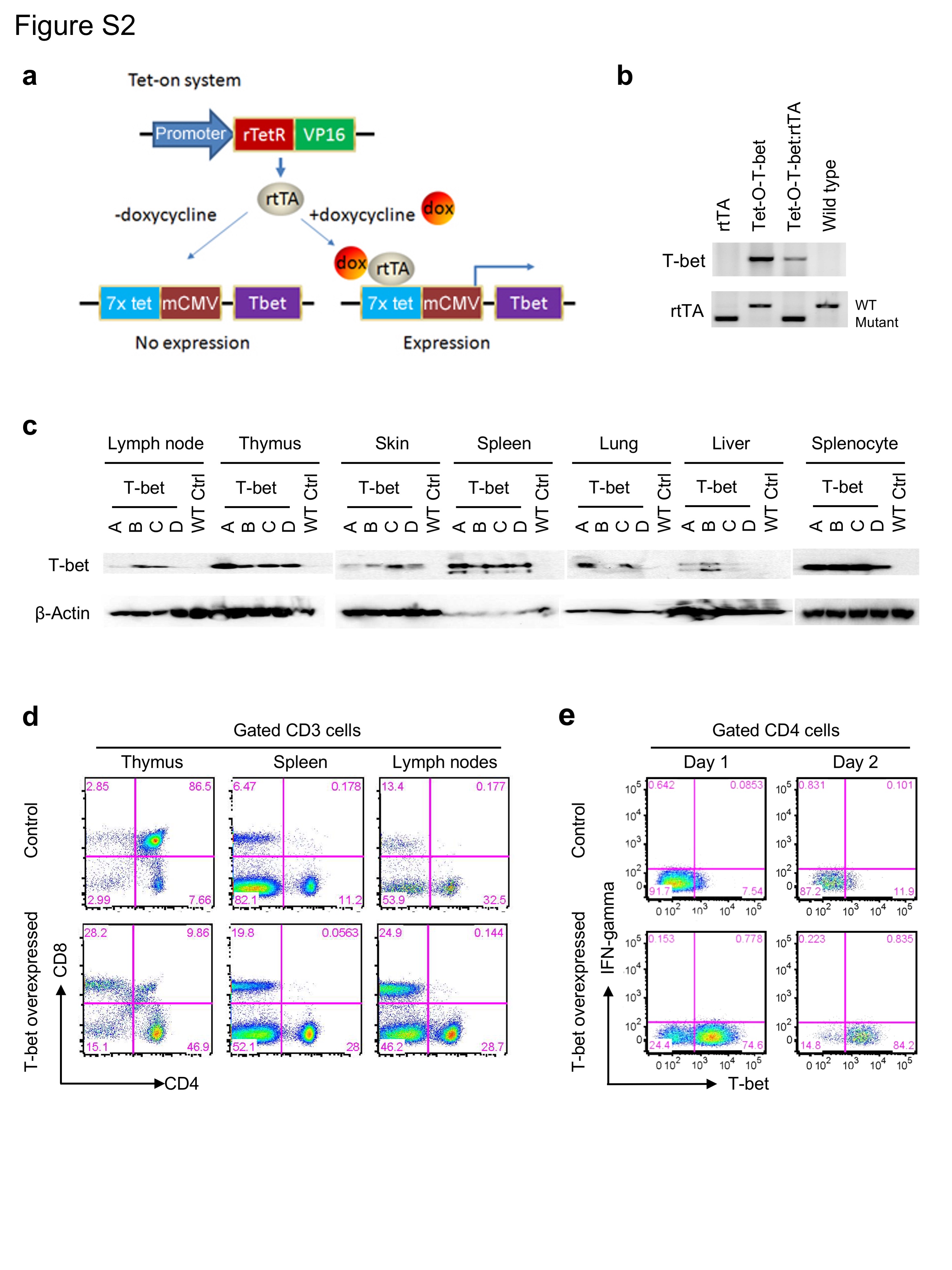
**

**Figure S2. Generation of Tet-O-T-bet:rtTA mice.**

(**a**) A schematic diagram of the Tet-On-induced T-bet expression system. Tet-O-T-bet transgenic mouse strain was generated by incorporating the mouse *Tbx21* coding sequence into a doxycycline-inducible expression system. To construct the tet response element (TRE), 7 repeats of tetracycline operator sequence (7× tet) were fused to a minimal cytomegalovirus (CMV) promoter (mCMV) which lacks an enhancer sequence. The expression of reverse tetracycline-controlled transactivator (rtTA) was controlled by a constitutive promoter. Without doxycycline (Dox), rtTA exhibits a reverse phenotype and cannot bind to the Tet-On sequences. The transcription complex is not assembled, and gene expression is silenced. In the presence of doxycycline, rtTA binds to the Tet-On sequences and recruits RNA polymerase II factors for the assembly of transcription complexes, initiating the transcription of T-bet gene.

(**b**) Genotyping strategy to identify the Tet-O-T-bet:rtTA transgenic mouse.

(**c**) Inducible expression of T-bet in major tissues and organs by western blotting. Four Tet-O-T-bet:rtTA mice and one WT mouse were treated with doxycycline water solution (1 mg/ml in 5% sucrose) for 2 days before tissue collection.

(**d**) Tet-O-T-bet:rtTA mice were treated with control water or doxycycline water solution for 2 days. Thymus, spleen, and lymph nodes were collected for flow cytometry analyses to detect the T cell differentiation in T-bet-overexpressed mice. The percentages of CD4^+^ T cells in thymus, spleen, and lymph nodes were 7.66%, 11.2%, and 32.5% in control mice, and 46.9%, 28%, and 28.7% in T-bet overexpressed mice, respectively. The percentages of CD8^+^ T cells in these organs were 2.85%, 6.47%, and 13.4% in control mice, and 28.2%, 19.8%, and 24.9% in T-bet overexpressed mice, respectively.

(**e**) Tet-O-T-bet:rtTA mice were treated with control water or doxycycline water solution. At days 1 and 2, splenocytes were harvested for flow cytometry analyses to examine T-bet and IFN-γ expression in gated CD4 cells. The percentages of T-bet^+^ CD4^+^ T cells in control vs T-bet overexpressed mice were 7.54% vs 74.6% on day 1, and 11.9% vs 84.2% on day 2, respectively.

**
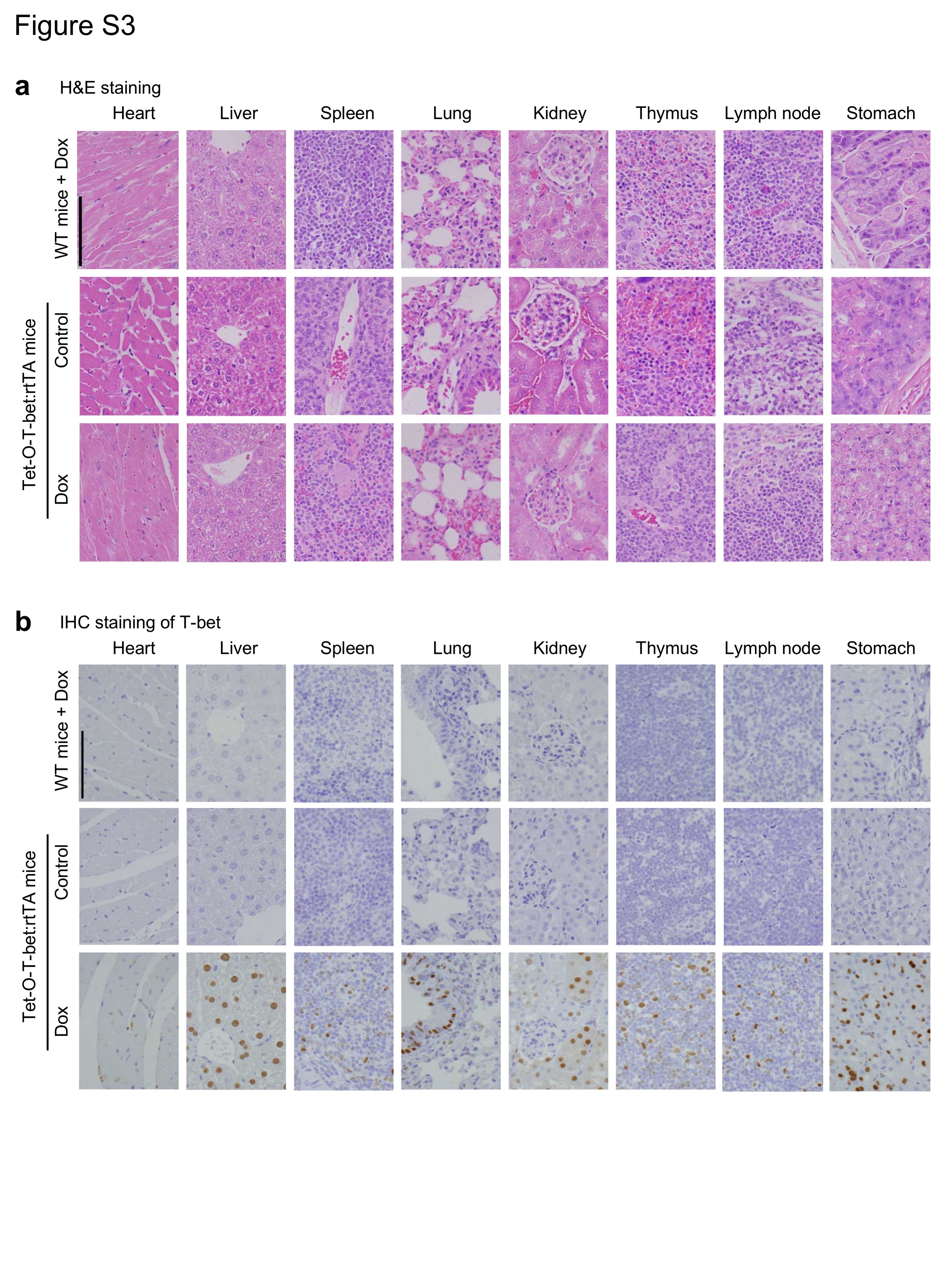
**

**Figure S3. Tissue structures and T-bet inducible expression in doxycycline-treated Tet-O-T-bet:rtTA mice.**

**(a**-**b**) WT and Tet-O-T-bet:rtTA (global T-bet expression) mice were treated with control water (5% sucrose alone) or doxycycline water solution (1 mg/ml in 5% sucrose) for 2 days. Major tissues and organs were collected for sectioning and histopathological analyses. (**a**) H&E staining to show tissue structure and potential damage in control and T-bet overexpressed mice (scale bar: 200 µm). (**b**) IHC staining of T-bet to validate the Dox-induced T-bet overexpression in different mouse tissues (scale bar: 200 µm).

**
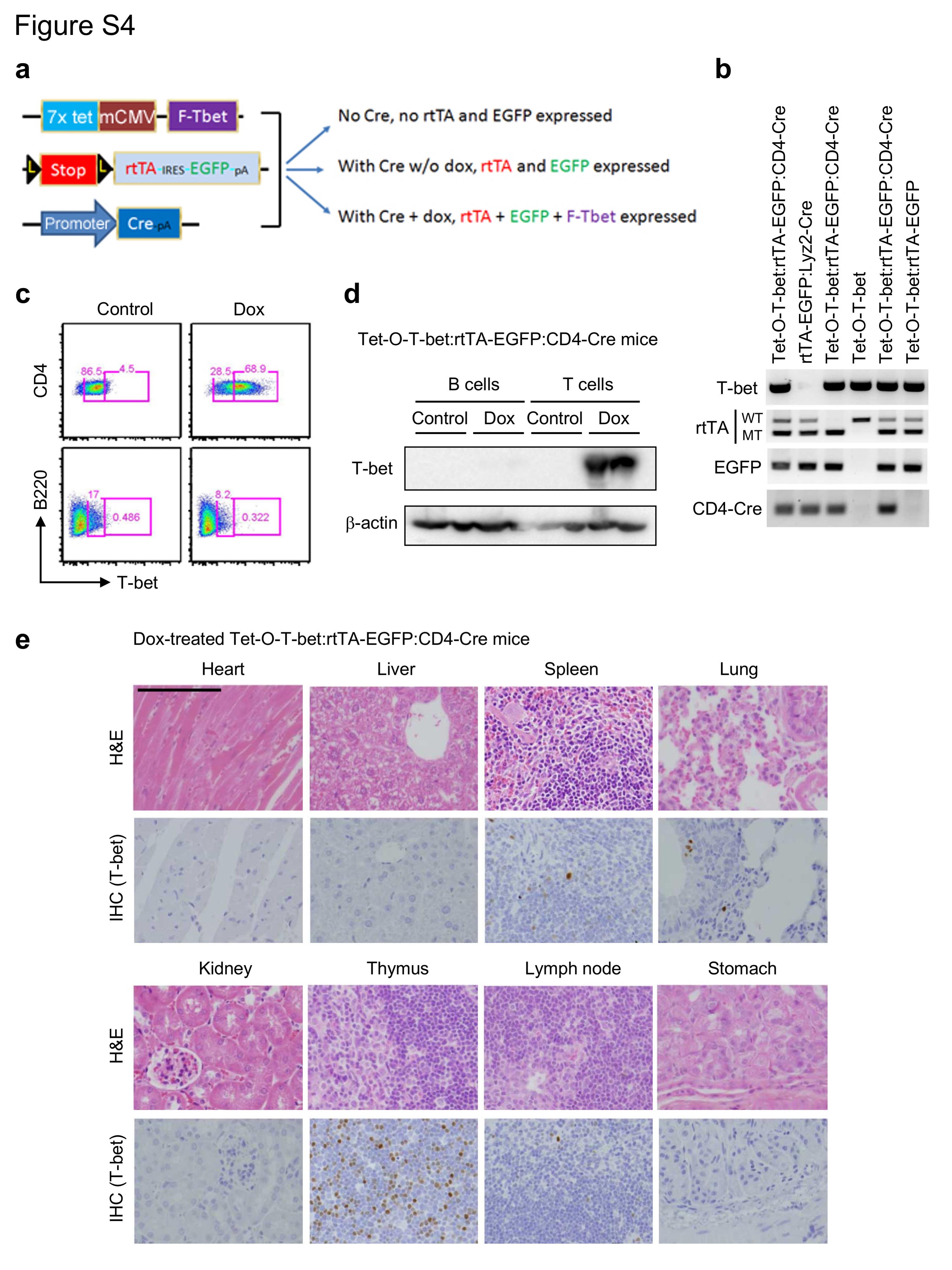
**

**Figure S4. Inducible expression of T-bet in CD4^+^ T cells does not cause mouse mortality.**

(**a**) A schematic diagram of tissue-specific doxycycline-inducible T-bet expression system. The LoxP-sites-flanked Stop sequence was inserted in front of EGFP-tagged rtTA. After crossing with specific Cre mice, rtTA can be expressed in specific tissues following Cre expression. In the presence of Dox, rtTA will bind to the Tet-On sequences, and T-bet expression will be activated in the specific Cre-expressing tissues.

(**b**) Genotyping strategy to identify the Tet-O-T-bet:rtTA-GFP:CD4-Cre transgenic mouse.

(**c**-**d**) CD4^+^ T cells and B220^+^ B cells were purified and sorted from spleens of Tet-O-T-bet:rtTA-GFP:CD4-Cre mice, then treated with PBS control or doxycycline (1 µg/ml) for 24 hours. (**c**) Flow cytometry analyses to validate the T-bet expression in gated CD4^+^ T cells. The percentages of T-bet^+^ CD4^+^ T cells are 4.5% in control mice and 68.9% in doxycycline-treated mice. B220^+^ B cells were tested as the negative control, in which the T-bet^+^ cells are 0.486% and 0.322% in control and doxycycline-treated mice, respectively. (**d**) Western blotting to detect the inducible T-bet expression in sorted CD4^+^ T cells.

(**e**) Tet-O-T-bet:rtTA-GFP:CD4-Cre mice were treated with control water (5% sucrose alone) or doxycycline water solution (1 mg/ml in 5% sucrose) for 2 days. H&E staining and IHC staining of T-bet on tissue sections from major organs to show tissue structure and T-bet overexpression levels (scale bar: 200 µm).

**
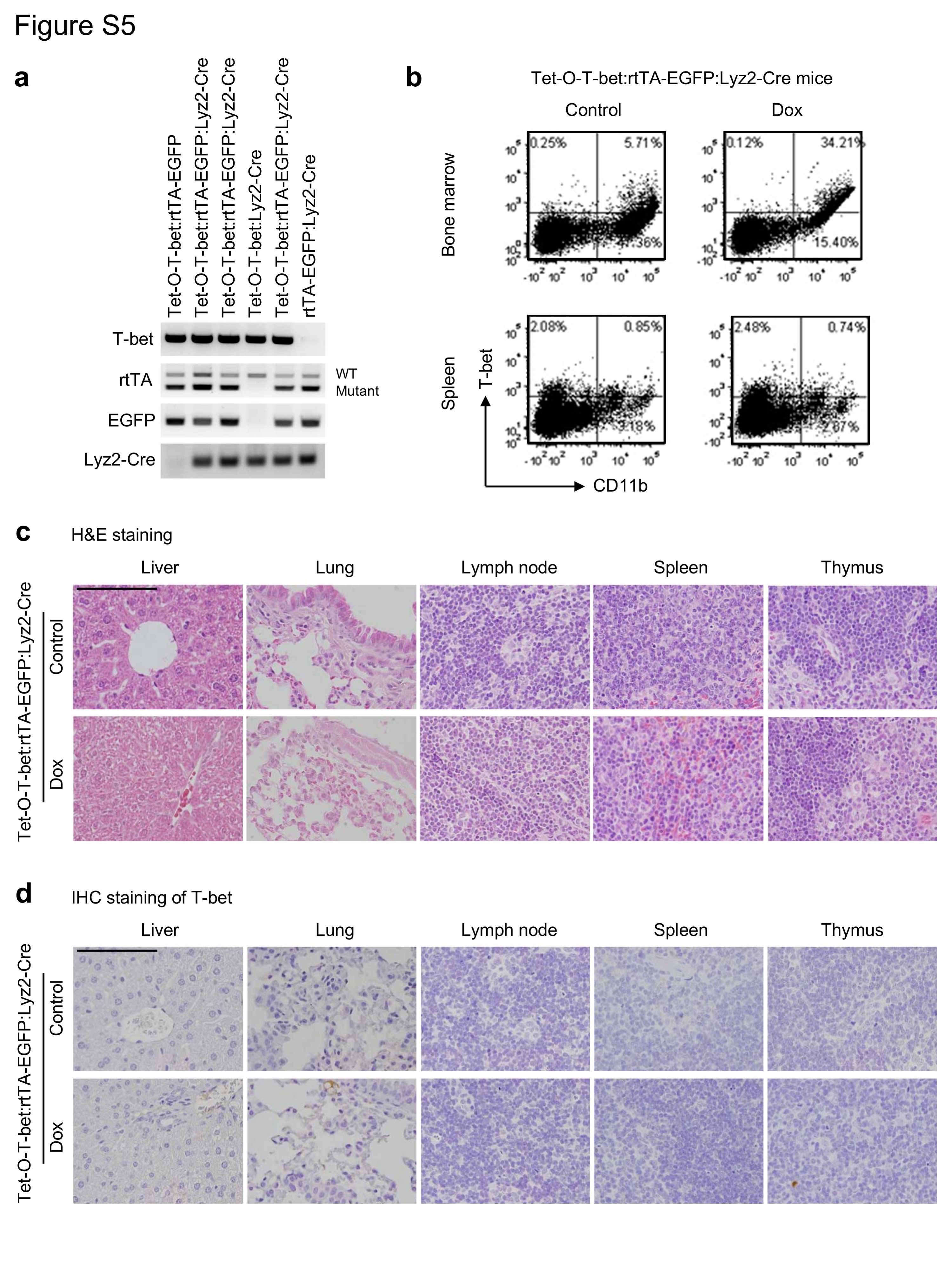
**

**Figure S5. Inducible expression of T-bet in myeloid cells does not cause mouse mortality.**

(**a**) Genotyping strategy to identify the Tet-O-T-bet:rtTA-GFP:Lyz2-Cre transgenic mouse.

(**b**-**d**) Tet-O-T-bet:rtTA-GFP:Lyz2-Cre mice were treated with control water (5% sucrose alone) or doxycycline water solution (1 mg/ml in 5% sucrose) for 2 days. (**b**) Spleen and bone marrow were collected for flow cytometry analyses to validate the T-bet expression in CD11b^+^ myeloid cells. In bone marrow, the percentages of T-bet^+^ CD11b^+^ myeloid cells are 5.71% in control mice and 34.21% in doxycycline-treated mice. On the contrary, in the spleen where lymphocytes are dominant, the percentages of T-bet^+^ CD11b^+^ cells are 0.85% and 0.74% in control and doxycycline-treated mice, respectively. (**c**) H&E staining on tissue sections from major organs to show tissue structure (scale bar: 200 µm). (**d**) IHC staining of T-bet on tissue sections for T-bet overexpression levels (scale bar: 200 µm).

**
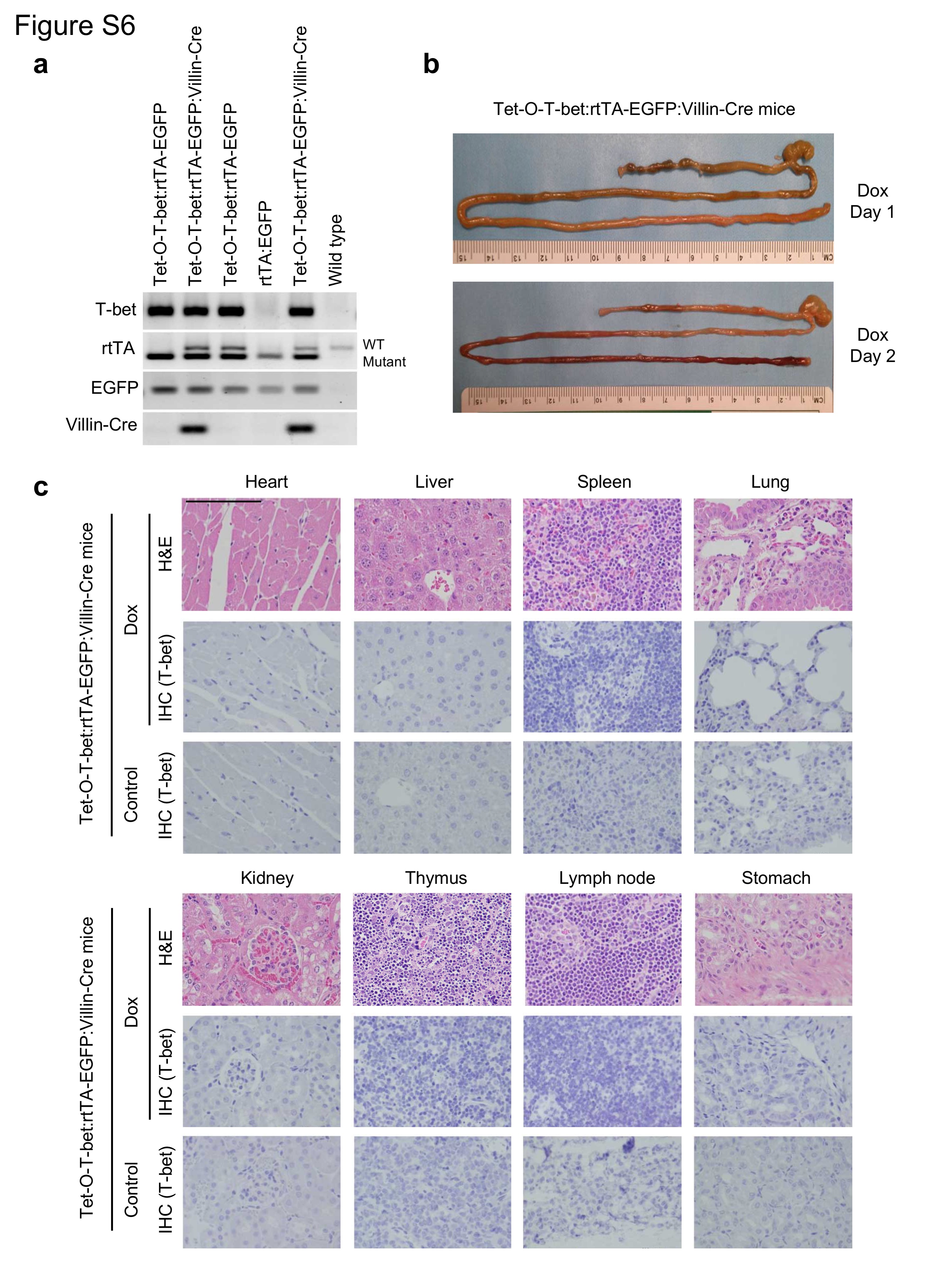
**

**Figure S6. Inducible expression of T-bet in gut epithelial cells causes mouse mortality.**

(**a**) Genotyping strategy to identify the Tet-O-T-bet:rtTA-GFP:Villin-Cre transgenic mouse.

(**b**-**c**) Tet-O-T-bet:rtTA-GFP:Villin-Cre mice were treated with control water (5% sucrose alone) or doxycycline water solution (1 mg/ml in 5% sucrose) for 2 days. (**b**) Mice were euthanized on days 1 and 2. Whole intestines were dissected to display the morphology. (**c**) Mice were euthanized on day 2 for tissue collection. H&E staining and IHC staining of T-bet on tissue sections from major organs (other than the intestine) to show tissue structure and T-bet expression levels (scale bar: 200 µm).

**
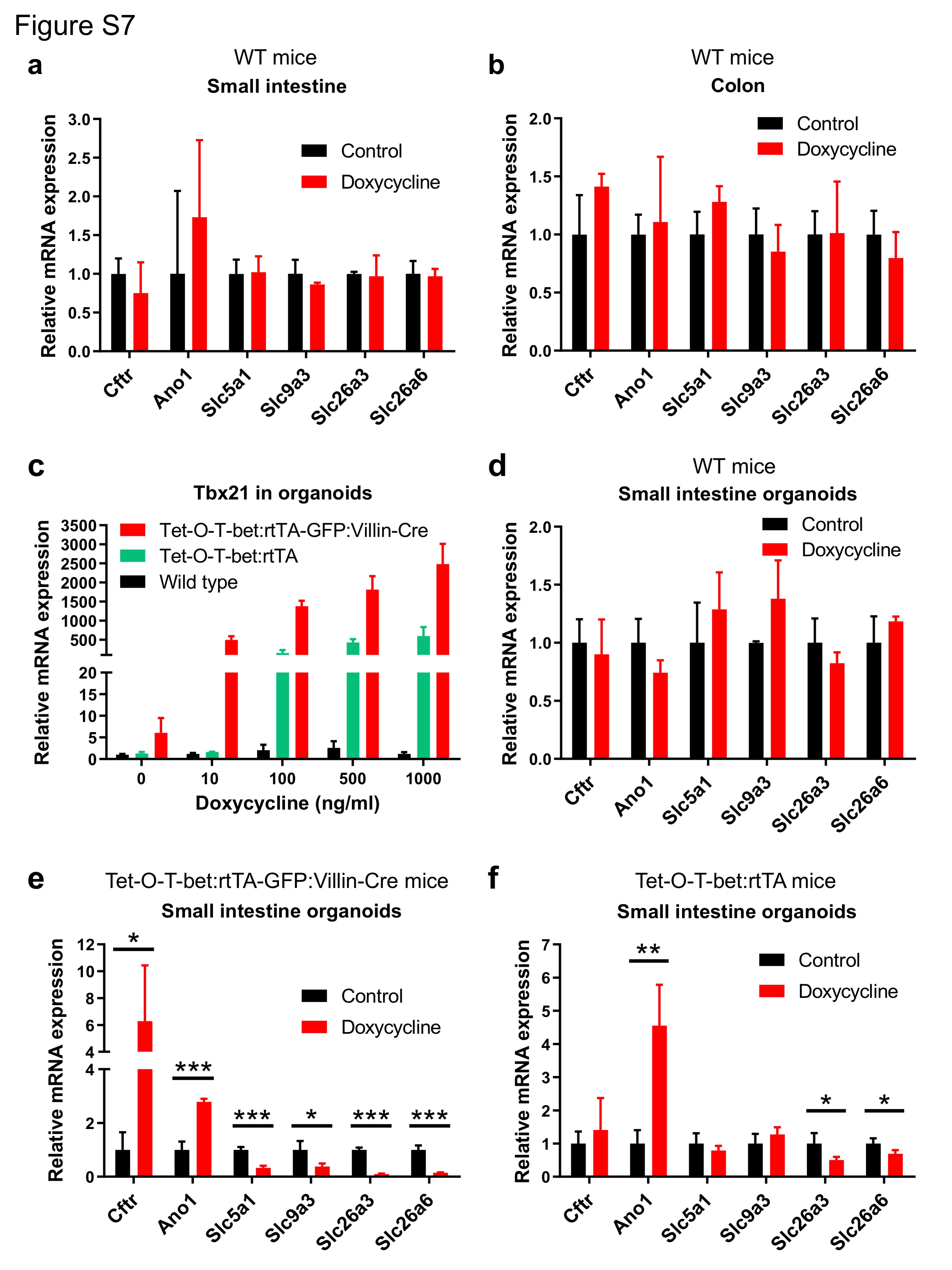
**

**Figure S7. T-bet regulates the expression of ion channels and transporters in mouse intestine.**

(**a**-**b**) WT control mice were treated with control water (5% sucrose alone) or doxycycline water solution (1 mg/ml in 5% sucrose) for 2 days. Mice were euthanized on day 2, and RNA samples were isolated from intestinal tissues. Real-time QPCR was performed to detect the expression levels of key ion channels and transporters in the small intestine (**a**) and colon (**b**) (mean ± SD).

(**c**-**f**) Small intestines were dissected from WT, Tet-O-T-bet:rtTA (global T-bet overexpression), and Tet-O-T-bet:rtTA-GFP:Villin-Cre (gut epithelia specific T-bet overexpression) mice, and the organoids were cultivated to recapitulate the *in vivo* tissue characteristics. (**c**) After the culture of 6-8 days, mature organoids were treated with different doses of doxycycline for 24 hours, and T-bet expression levels were examined by Real-time QPCR with purified RNA samples. Next, the organoids were treated with 1 µg/ml doxycycline for 24 hours, and RNA samples were isolated. Real-time QPCR was performed to detect the key ion channels and transporters in organoids from WT (**d**), Tet-O-T-bet:rtTA-GFP:Villin-Cre (**e**), and Tet-O-T-bet:rtTA (**f**) mice (mean ± SD).

Statistical analyses: Student’s unpaired t test (**a-b**, **d-f**). *p<0.05; **p<0.01; ***p<0.001.

**
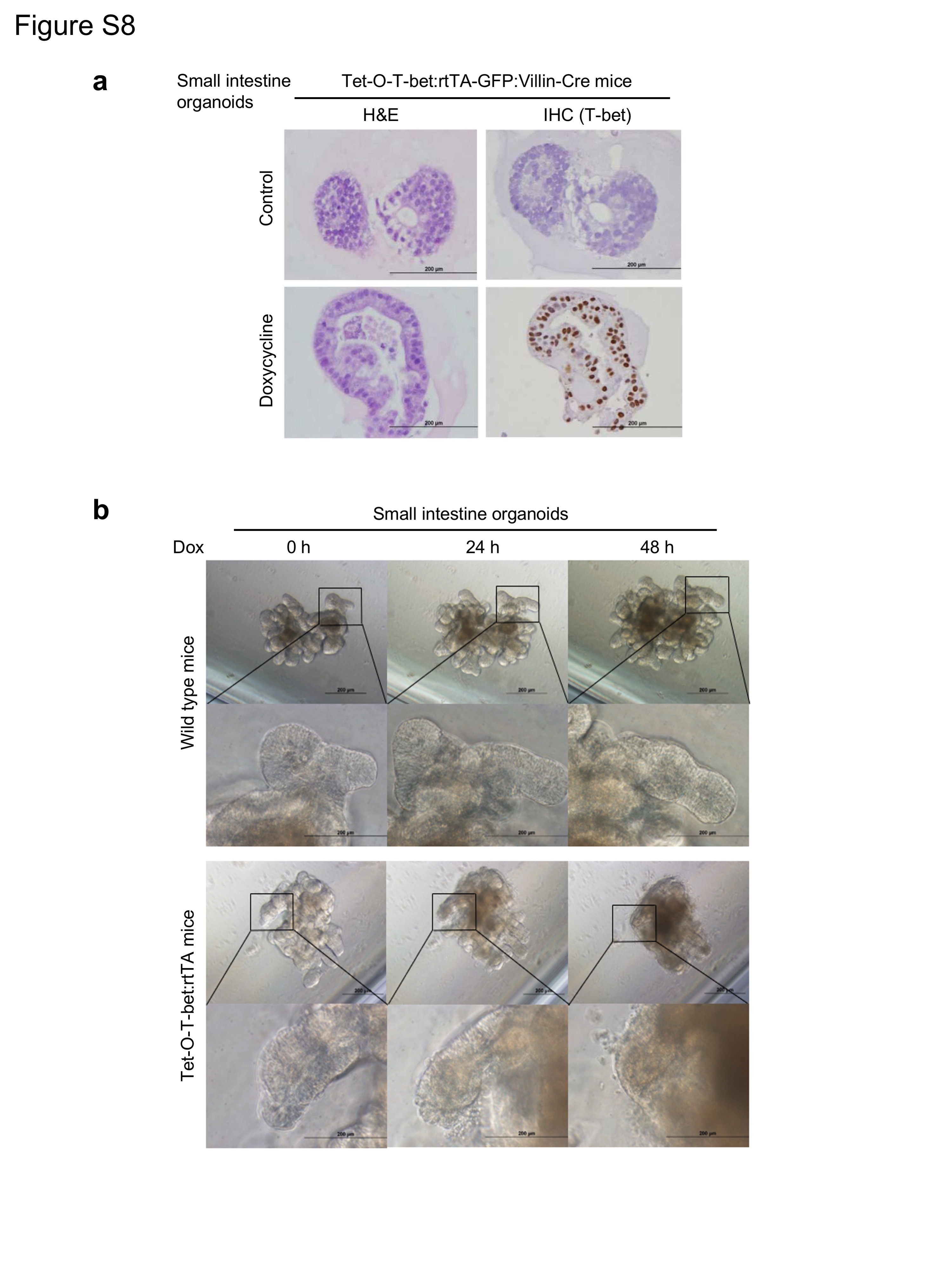
**

**Figure S8. Inducible T-bet expression promotes the apoptosis of intestinal epithelial cells.**

(**a**) Small intestines were dissected from Tet-O-T-bet:rtTA-GFP:Villin-Cre (gut epithelia specific T-bet overexpression) mice. Tissue organoids were cultivated and treated with PBS control or 1 µg/ml doxycycline for 24 hours. Then the organoids were fixed with formalin for 1 day and processed for sectioning. H&E staining and IHC staining of T-bet were performed to show the structure and T-bet expression in the organoids (scale bar: 200 µm).

(**b**) Small intestines were dissected from WT and Tet-O-T-bet:rtTA (global T-bet overexpression) mice, and the organoids were cultivated and treated with 1 µg/ml doxycycline. The morphological changes of these organoids were observed at different time points under the microscope (scale bar: 1 mm for low magnification and 200 µm for high magnification).

**
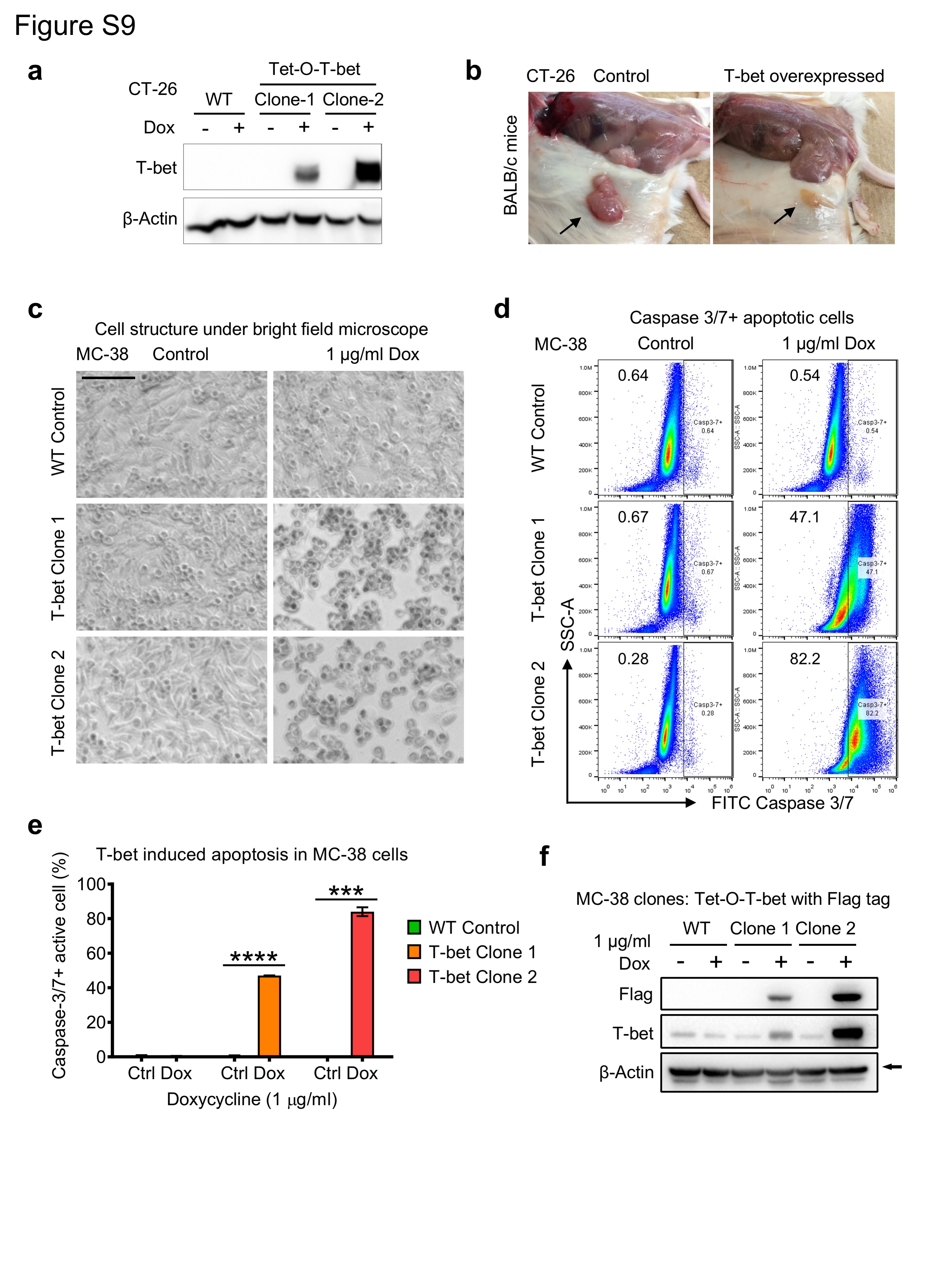
**

**Figure S9. Inducible T-bet expression completely inhibits colon tumor formation and growth.**

(**a**) WT control and Tet-O-T-bet transduced CT-26 single clones were treated with PBS control or 1 µg/ml doxycycline for 12 hours. Western blotting was performed to validate the Dox-induced T-bet expression.

(**b**) WT control and Tet-O-T-bet transduced CT-26 cells were subcutaneously (s.c.) injected into WT BALB/c mice, and continuously treated with doxycycline water solution (1 mg/ml in 5% sucrose) since day 7. The images of tumors on day 15 to compare the tumor sizes between two groups.

(**c-e**) WT control and Tet-O-T-bet transduced MC-38 cells were treated with PBS control or 1 µg/ml doxycycline for 3 days. (**c**) The morphological change in cells with Dox-induced T-bet overexpression was observed under the microscope (scale bar: 200 µm). (**d**) Flow cytometry to determine the apoptotic cell percentages (Caspase 3/7^+^) in MC-38 cell clones with Dox-induced T-bet overexpression. The percentages of Caspase 3/7^+^ cells before and after doxycycline treatment were 0.64% vs 0.54% in WT cells, 0.67% vs 47.1% in T-bet overexpressed clone 1, and 0.28% vs 82.2% in T-bet overexpressed clone 2, respectively. (**e**) Statistical analysis of the flow cytometry data in **d** (mean ± SD).

(**f**) WT control and Tet-O-T-bet transduced MC-38 single clones were treated with PBS control or 1 µg/ml doxycycline for 24 hours. Western blotting was performed to validate the Dox-induced T-bet expression.

Statistical analyses: Student’s unpaired t test (**e**). ***p<0.001; ****p<0.0001.
